# A promising Prognostic risk model for advanced renal cell carcinoma (RCC) with immune-related genes

**DOI:** 10.1186/s12885-022-09755-2

**Published:** 2022-06-23

**Authors:** Peng Cao, Ji-Yue Wu, Jian-Dong Zhang, Ze-Jia Sun, Xiang Zheng, Bao-Zhong Yu, Hao-Yuan Cao, Fei-Long Zhang, Zi-Hao Gao, Wei Wang

**Affiliations:** Department of Urology, Beijing Chaoyang Hospital, Capital Medical University, Beijing, China

**Keywords:** Renal cell carcinoma, RCC, Immunologic signature, Tumor immunity, Prognosis

## Abstract

**Background:**

Renal cell carcinoma (RCC) is a third most common tumor of the urinary system. Nowadays, Immunotherapy is a hot topic in the treatment of solid tumors, especially for those tumors with pre-activated immune state.

**Methods:**

In this study, we downloaded genomic and clinical data of RCC samples from The Cancer Genome Atlas (TCGA) database. Four immune-related genetic signatures were used to predict the prognosis of RCC by Cox regression analysis. Then we established a prognostic risk model consisting of the genes most related to prognosis from four signatures to value prognosis of the RCC samples via Kaplan–Meier (KM) survival analysis. An independent data from International Cancer Genome Consortium (ICGC) database were used to test the predictive stability of the model. Furthermore, we performed landscape analysis to assess the difference of gene mutant in the RCC samples from TCGA. Finally, we explored the correlation between the selected genes and the level of tumor immune infiltration via Tumor Immune Estimation Resource (TIMER) platform.

**Results:**

We used four genetic signatures to construct prognostic risk models respectively and found that each of the models could divide the RCC samples into high- and low-risk groups with significantly different prognosis, especially in advanced RCC. A comprehensive prognostic risk model was constructed by 8 candidate genes from four signatures (HLA-B, HLA-A, HLA-DRA, IDO1, TAGAP, CIITA, PRF1 and CD8B) dividing the advanced RCC samples from TCGA database into high-risk and low-risk groups with a significant difference in cancer-specific survival (CSS). The stability of the model was verified by independent data from ICGC database. And the classification efficiency of the model was stable for the samples from different subgroups. Landscape analysis showed that mutation ratios of some genes were different between two risk groups. In addition, the expression levels of the selected genes were significantly correlated with the infiltration degree of immune cells in the advanced RCC.

**Conclusions:**

Sum up, eight immune-related genes were screened in our study to construct prognostic risk model with great predictive value for the prognosis of advanced RCC, and the genes were associated with infiltrating immune cells in tumors which have potential to conduct personalized treatment for advanced RCC.

**Supplementary Information:**

The online version contains supplementary material available at 10.1186/s12885-022-09755-2.

## Background

Renal cell carcinoma (RCC) is the 14^th^ most common cancer accounting for 2.2% of all cancers worldwide. 403,262 new cases have been reported in 2018 with a ratio of males to females being estimated as 1.5:1 [1]. RCC is not a single disease, but rather comprises a number of different types of tumors that arise from the renal epithelium [2]. It can be divided roughly into clear cell RCC (ccRCC) and non-clear-cell RCC (nccRCC). ccRCC is the most common subtype of RCC and accounts for > 80%. papillary RCC (pRCC), and chromophobe cell renal carcinoma (ccRC) are represented subtypes in nccRCC accounting for 10–15% and 4–5% of RCC, respectively [3]. Unclassified RCC represents 2–6% of renal tumours. Despite the diagnosis and the improved treatment of RCC, its overall survival remains low especially advanced RCC. The problem may be attributed to the lack of specific treatments for different subtypes of RCC and tumor progression. Most of available treatments focused on ccRCC. Additionally, the same methods would be used for nccRCC due to a lack of effective treatments for the disease. Surgery is the first choice for localized RCC. The standard treatment of localized RCC is the radical nephrectomy or partial nephrectomy. However, as the tumor progresses, its treatment and prognosis will become intractable. Data indicate that the age-standardized 5-year relative survival of RCC patients decreases with the increase in the clinical stage [4]. Cytoreductive nephrectomy (CN) could be performed for the advanced RCC and need to be supplemented with adjuvant therapy in the past cytokine therapy era. But it was worth noting that advanced RCC patients with poor physical condition couldn’t benefit from CN [5]. The systemic treatments are recommended therapies for advanced RCC or metastatic RCC [6, 7]. In addition, other treatments, such as embolization, targeted therapy and immunotherapy as a supplement or alternative for surgery, provide new visions for successful treatment and better prognosis of RCC [3, 8].

RCC is a malignant tumor which is insensitive to traditional radiotherapy and chemotherapy. It has strong immunogenicity and is considered as a hot tumor in which a large number of B cells, T cells, macrophages and other immune cells infiltrate the tumor tissue. Therefore, immunotherapy is a good choice for its treatment. At present, immunotherapy has leaped to the forefront of cancer research. Endless new immunotherapy drugs have been approved for a variety of solid tumors. In particular, the overall therapeutic effect of patients with advanced and metastatic RCC has improved in recent years [9, 10]. With the development of RCC genomic research and the new progress about the mechanism of immune response to cancers, the immunotherapy of RCC has shifted from non-specific immunotherapy (cytokine therapy) to new types of immunotherapy (immune checkpoint inhibitor, combined immunotherapy), which opens a new era of immunotherapy for RCC. For example, PD1/PD-L1, CTLA-4 and other immune checkpoints, which are negative costimulatory molecular control signals, inhibit the activation and function of T cells, and promote tumor immune escape and self-proliferation [11]. Immune checkpoint inhibitors block the immunomodulatory effect of these inhibitory immune checkpoints and indirectly strengthen the anti-tumor immune response and improve the therapeutic effect. However, the incidence of immune related adverse events (irAEs) in patients receiving immunosuppressive therapy was very high, up to more than 70% [12]. Therefore, it is highly prerequisite to discovery biomarkers which better assess advanced RCC prognosis and are correlated with the immune cells infiltrating in the hot tumor. This will help to provide potential targets of immunotherapy for advanced RCC and to curative effect.

Here, we have downloaded 758 different pathological types of RCC samples from the TCGA database, and used 4 reported immune-related genetic signatures to evaluate RCC prognosis. We have selected 8 candidate genes (*HLA-B*, *HLA-A*, *HLA-DRA*, *IDO1*, *TAGAP*, *CIITA*, *PRF1* and *CD8B*) from each signature and combined them to construct a comprehensive prognostic risk model that divides the advanced kidney cancer into high- and low-risk groups. We have detected that the cancer-specific survival (CSS) of the high-risk group was significantly lower than that of the low-risk group. After the verification of the model, we found that mutation ratios of some genes were different between two risk groups and there were some correlations between the expression of 8 genes and the degree of the tumor infiltrating immune cells.

## Materials and methods

### Data acquisition

Patients’ clinical information and mRNA expression profiles from The Cancer Genome Atlas (TCGA) database of three main pathologic types of RCC, i.e. kidney renal clear cell carcinoma (KIRC), kidney renal papillary cell carcinoma (KIRP) and kidney chromophobe (KICH) were downloaded from UCSC Xena. The R packet TCGA biolinks were used to obtain the genetic mutation information. (91 RCC samples used for validating the prognostic risk model came from International Cancer Genome Consortium (ICGC) database (Table 1).) Genes used for analysis in the present study were from four immune-related genetic signatures which were as follows: *HLA-A* and *HLA-B* in HLA class I molecules, IFN gamma signature, expended immune gene signature and cytotoxic T lymphocyte (CTL) level signature. The corresponding genes in the signatures were reported to be closely related to the clinical outcomes and prognosis of solid tumors (Table 2).

**Table 1:** Data of three main subtypes of RCC, kidney renal clear cell carcinoma (KIRC), kidney renal papillary cell carcinoma (KIRP) and kidney chromophobe (KICH), from TCGA and ICGC database

Data	Number of RCC sample
KICH	62
KIRC	429
KIRP	267
RCC from ICGC	91

**Table 2:** Immune-related signatures used in the study including HLA-A and -B, IFN gamma signature, expanded immune gene signature, and cytotoxic T lymphocyte (CTL) signature, and their corresponding genes

Signature	Gene
HLA class I molecules	HLA-A, HLA-B
IFN gamma signature	IDO1, CXCL10, CXCL9, HLA-DRA, IFNG
Expanded immune gene signature	CD30(TNFRSF8), IDO1, CIITA, CD3E, CCL5, GZMK, CD2, HLA-DRA, CXCL13, NKG7, HLA-E, CXCR6, LAG3, TAGAP, CXCL10, STAT1, GZMB
Cytotoxic T lymphocyte (CTL) level signature	CD8A, CD8B, GZMA, GZMB, PRF1

### Survival analysis via univariate COX regression analysis

The survival time and survival status of the patients with RCC were extracted from TCGA database. And the samples with incomplete clinical data were removed. Taken together, a total number of 730 samples with complete prognostic outcome were selected. According to the clinical stage of the tumor, recommended by the American Joint Committee on Cancer (AJCC) [13], all samples were divided into two groups that comprised four stages. The first group comprised stages I and II and was designated as an early stage RCC group while the second group was the advanced RCC group and included RCC in stages III and IV. We used the coxph function in R packet survival to conduct univariate COX regression analysis and to explore the association between the corresponding genes in each immune-related genetic signature and the disease-free survival (DFS) and overall survival (OS) of the two groups of RCC samples.

### Establishment and validation of the prognostic model

Genes in the signatures for multiple COX regression analysis constructed four prognostic risk models for early and advanced RCC, respectively. The two genes most related to the prognosis in the advanced RCC group were selected from each of the signatures. And the selected 8 genes were used for multiple COX regression analysis to construct a new comprehensive prognostic risk model. Then the surv_cutpoint function in R packet survminer was applied to determine the best threshold point to distinguish between the low-risk and high-risk RCC. Kaplan–Meier (KM) survival analysis was used to evaluate the predictive ability of the prognostic model. We then built receiver operating characteristic (ROC) curves to evaluate the specificity and sensitivity of the model via survival ROC in R packet. And the prognosis risk model was tested by independent data obtained from ICGC database. Last but not the least, we have applied the model to different clinical subtypes, such as age, gender, clinical stage and pathological pattern, to assess its stability.

### Gene mutation analysis of the prognostic model based on TCGA database

R packet maftools have been used to calculate the gene mutations for each patient with RCC genetic data in TCGA database. We have screened 16 genes in low- and high-risk samples, respectively according to the mutation ratio, and then built a waterfall map to show the distribution of the mutations of the genes in the two groups of RCC samples. The mutation information of immune-related genes in our model in the TCGA RCC dataset were acquired from the cBioPortal database.

### Association among the tumor infiltrating immune cells and selected genes

Tumor Immune Estimation Resource, TIMER [14] is a comprehensive database to study tumor infiltrating immune cells in various malignancies systematically. The web contains a large number of different cancer samples in TCGA database. We have investigated the association of six types of immune infiltrating cells (B cells, CD8 + T cells, CD4 + T cells, macrophages, neutrophils, and dendritic cells) with 8 selected genes to evaluate the immune status of the tumor in the low and high-risk groups via TIMER platform.

### Statistical analysis

All statistical analysis was carried out in R studio (version 3.6.2). R packet survival and survminer were used to perform univariate/multivariate Cox analysis, Kaplan–Meier survival analysis and determine optimal threshold points to classify low-risk and high-risk renal cancer patients. R packet maftools, which was used for statistical analysis of gene mutations in renal cancer patients. R packet pheatmap was used for drawing heatmap in the analysis. R package survivalROC is used to draw the ROC curve. *P* value < 0.05 was considered statistical significance.

## Results

### Association of genes in described immune-related signatures with disease free survival and overall survival in RCC

In our present study, we downloaded the data of 758 RCC samples from TCGA database and independent data of 91 RCC samples from ICGC database, respectively. The analysis of the correlation between the expression levels of immune-related genes and the prognosis of RCC allowed us to select genes derived from IFN-gamma signature, extended immune gene signature, cytotoxic T lymphocyte (CTL) signature and HLA-A and HLA-B in HLA I molecules. These immune-related signatures were reported to be related to the prognosis of solid tumors, such as melanoma, ovarian cancer, breast cancer [15–19].

The univariate COX regression analysis was used to correlate gene expression levels with DFS and the OS of RCC. First, according to the clinical stage, we have divided the samples into two groups: an early-stage group that comprised RCC in stages I and II and an advanced stage group containing RCC in stages III and IV. After excluding the invalid samples, 499 early RCC and 231 advanced RCC samples were further analyzed. In the two groups of RCC subsets, we found that a few of immune-related genes were significantly associated with DFS and OS of RCC patients. For the early stage RCC, we found that high expression levels of *CXCL13* and *STAT1* resulted in poor DFS while the high expression levels of *IDO1*, *CXCL13* and *GZMB* were related to detrimental OS. For the advanced RCC, the high expression levels of *TNFRSF8* and *CXCL13* were shown to be good predictors of adverse DFS and OS, respectively. (Supplementary Fig. 1).

### Construction of prognostic risk models of RCC on the basis of genes from each immune-related signature

**Fig. 1:**
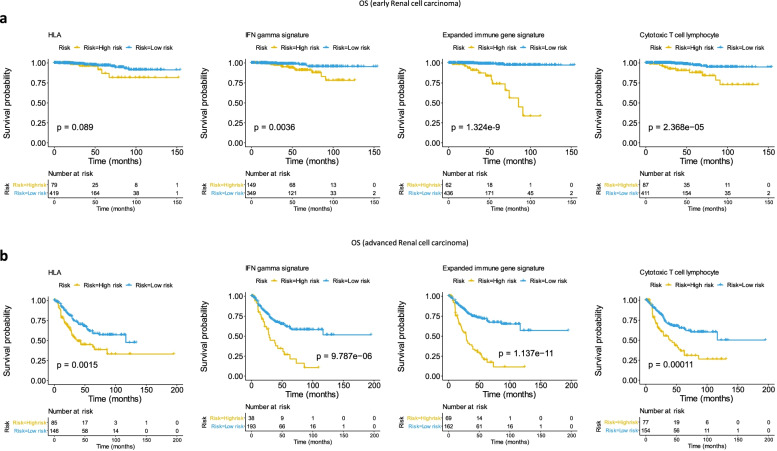
Prognostic risk models constructed by four immune-related signatures for overall survival (OS) in early and advanced RCC. **A** Classified efficiency of prognostic risk models constructed by four immune-related signatures (IFN-gamma signature, extended immune gene signature, cytotoxic T lymphocyte signature and HLA-A and HLA-B) in stage I + II RCC. **B** Classified efficiency of the four prognostic risk models for stage III + IV RCC. The p-value was shown in the survival plots

Genes from four gene signatures were studied to perform a multiple COX regression analysis in early and advanced RCC groups and to construct prognostic models for the OS of RCC and to evaluate the performance of each model in the two groups of samples. The model was used to calculate the risk score of each sample. It determined the division threshold according to the surv_cutpoint function, divided the samples into high-risk and low-risk groups, and conducted the KM survival analysis according to the high and low risk groups of the samples. All four models allowed discrimination of the RCC samples into high and low risk groups. The OS was worse in the high-risk RCC group than in the low-risk one. Contrary to the early stage RCC, the survival curves for the four models of immune-related signatures indicated more significant differences in the OS between the two groups from the advanced RCC (HLA-A and HLA-B: *p* value = 0.0015; IFN-gamma signature: *p* value = 9.787e-6; Expended immune gene signature: *p* value = 1.137e-11; Cytotoxic T cell lymphocyte signature: *p* value = 0.00011) as shown in Fig. 1.

### Establishment and examination of the prognostic model with selected genes for advanced RCC

**Fig. 2:**
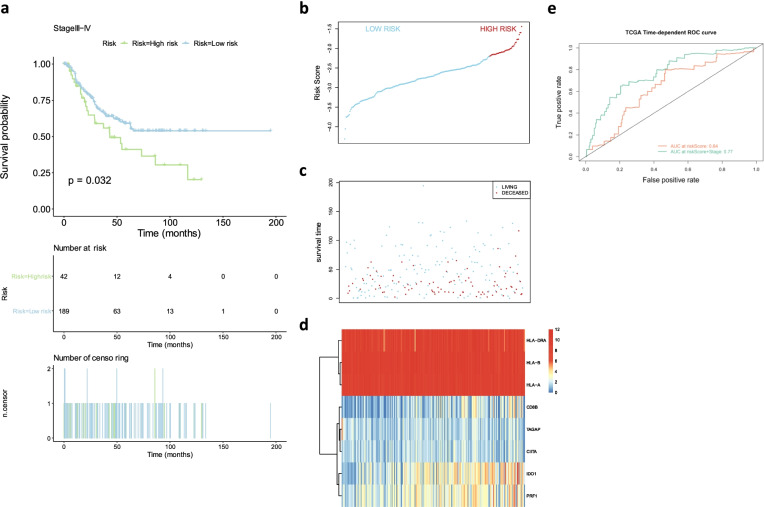
Prognostic risk models constructed by 8 genes combination for OS in advanced RCC. **A** Survival plots showed OS of high-risk group and low-risk group in advanced RCC. The risk score curve **B** and the scatter plot **C** were drawn according to risk score of every advanced RCC sample calculated by the model. **D** The heatmap indicated the expression levels of selected genes in the advanced RCC samples. High and low expressions were highlighted in red and blue, respectively. **E** The predicted value of the model was assessed by Time-dependent ROC curve. The p-value was shown in the survival plot

The four risk models constructed by using the four immune-related signatures in advanced RCC samples divided the samples into high and low risk groups with significant statistical differences in the overall survival rate. Therefore, we have used 8 genes that were most likely to be associated with cancer-specific survival (CSS) in the advanced RCC samples. These genes were *HLA-A*; *HLA-B*; *HLA-DRA*; *IDO1*; *TAGAP*; *CIITA; PRF1* and *CD8B*. These genes were combined to make multiple COX regression analysis, and a comprehensive prognostic risk model was constructed according to expression of the genes and their gene weight coefficient. Concretely speaking, the risk score is the sum of the amount of each gene expression multiplied by weight coefficient of the gene in the model (see Supplementary Table 1). The advanced RCC samples could also be divided into high-risk and low-risk groups according to risk score of each sample (Supplementary Table 2) and the division threshold, cutoff = -2.20465, showing in Fig. 2B. The CSS in the high-risk group is lower than that in the low-risk group and there were significant differences in the CSS between the two groups with *p* value = 0.032 (Fig. 2A). The ROC curve suggested that the comprehensive prognostic risk model built on the data for the studied 8 genes was relatively stable for survival prognosis prediction of advanced RCC (area under curve (AUC) = 0.64 showing in the Fig. 2E).

**Fig. 3:**
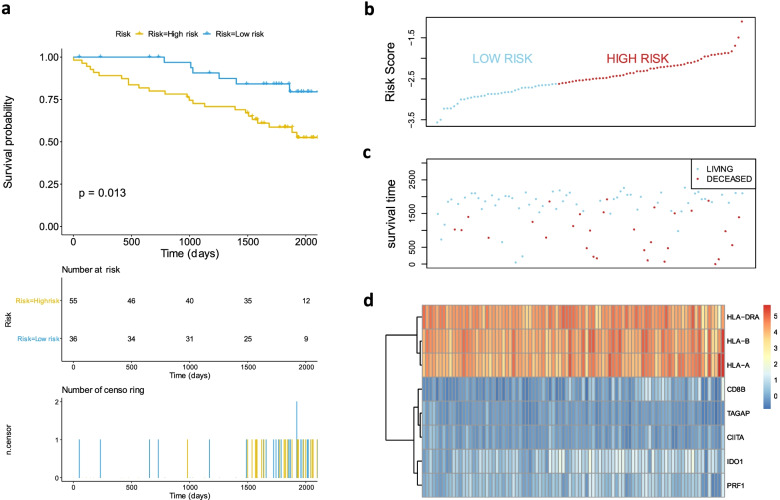
Validating the classified efficiency of the prognostic risk model constructed by 8 selected genes combination via data from ICGC. **A** Survival plots showed OS of high-risk group and low-risk group in advanced RCC from ICGC. The risk score curve **B** and the scatter plot **C** were drawn according to risk score of each RCC sample calculated by the model. (**D**) The heatmap indicated the expression levels of selected genes in the RCC samples. The *p*-value was shown in the survival plot

We further used 91 of RCC samples from the ICGC database to test the comprehensive prognostic risk model. The division threshold determined by the above method was -2.622015 (Fig. 3B). And the samples were divided into high and low risk groups (Supplementary Table 3) according to the threshold. The results showed that the CSS in the high-risk group was significantly lower than that in the low-risk group (*p* value = 0.013 showing in the Fig. 3A). The prediction result of the model was consistent with the previous results (Fig. 2 and Fig. 3), and the stability of the model was effectively verified.

### Gene mutation analysis

**Fig. 4:**
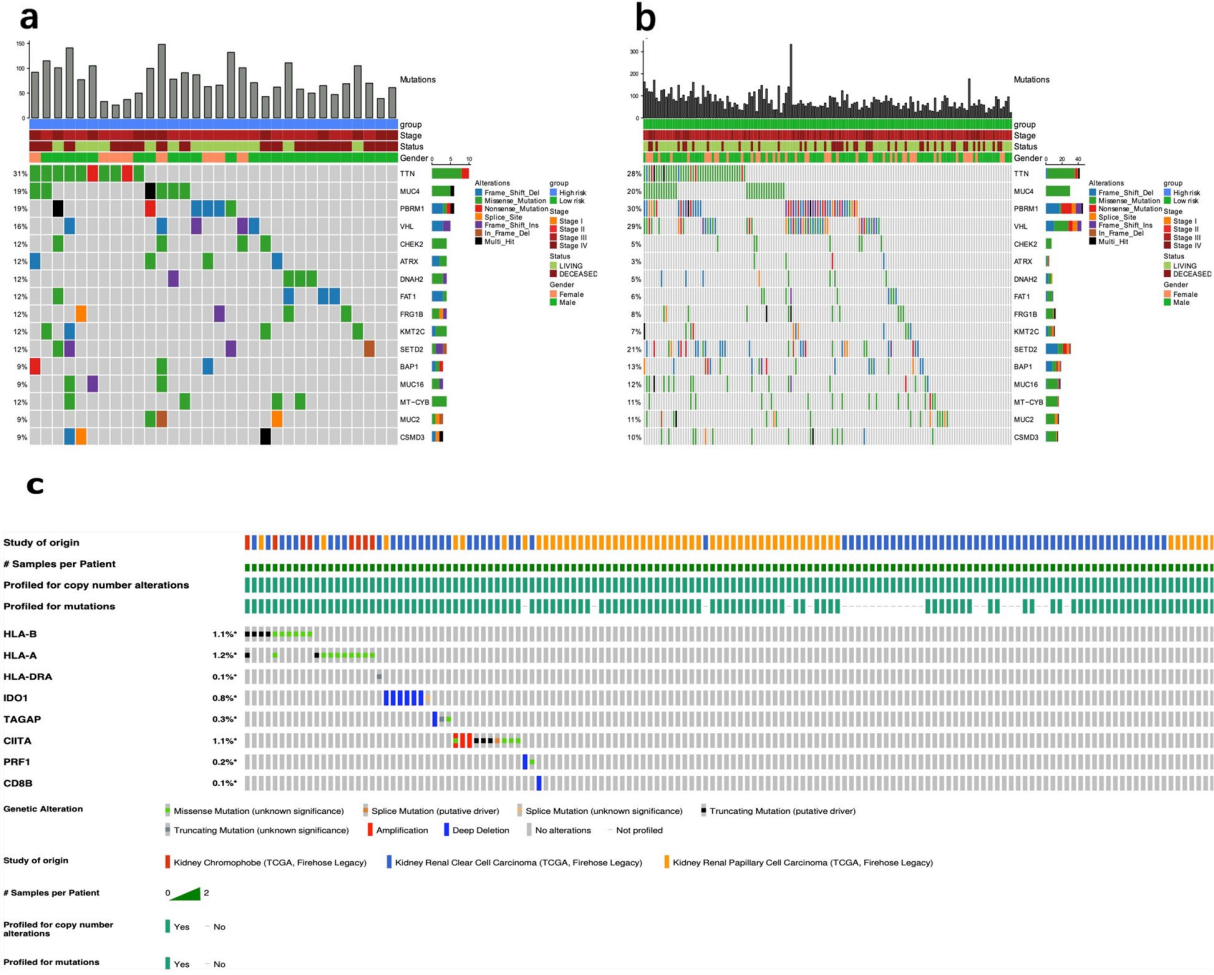
Gene mutation analysis. The landscape analysis showed the top 16 genes with mutation frequency in high-risk group **A** and low-risk group **B **of the advanced RCC. The histogram showed the number of mutations in the RCC samples. Annotation information of the samples included risk groups, clinical stages, living status and genders. Different colors represented different mutation types. **C** Genetic alterations of the 8 selected genes in RCC samples

Landscape analysis of gene mutation showed that there were many kinds of mutations in different genes of the advanced RCC samples (see Supplementary table 5) from the TCGA database. Among the advanced RCC samples, the genes with the top 10 mutation rates in the high risk group included *TTN*, *MUC4*, *PBRM1, VHL, CHECK2, ATRX, DNAM2, FAT1, FRG1B, KMT2C* (Fig. 4A), while in the low risk group these genes were *PBRM1*, *VHL, TTN, SETD2, MUC4, BAP1, MUC16, MT-CYB, MUC2, CSMD3* (Fig. 4B). The distribution and annotation of mutations of top16 mutant genes in the two groups of samples showed in the Fig. 4A-B. The frequencies of the mutant genes, such as *VHL*, *CHEK2 and ATRX,* were different between the high-risk and low-risk groups. Among them, the frequency of *ATRX* in the high-risk group was significantly higher than that in the low-risk group (*p* value = 0.0455).

As we all know, molecular events such as gene amplification, mutation, and deletion may affect gene expression. Therefore, we further analyzed the mutations of immune-related genes in our model. As for the RCC patient cohort in the TCGA database, genetic alteration percentages of these immune-related genes ranged from 0.1% to 1.2%, which indicated that these mutations have little effect on gene expression (Fig. 4C).

### Stability assessment of prognostic risk model

**Fig. 5:**
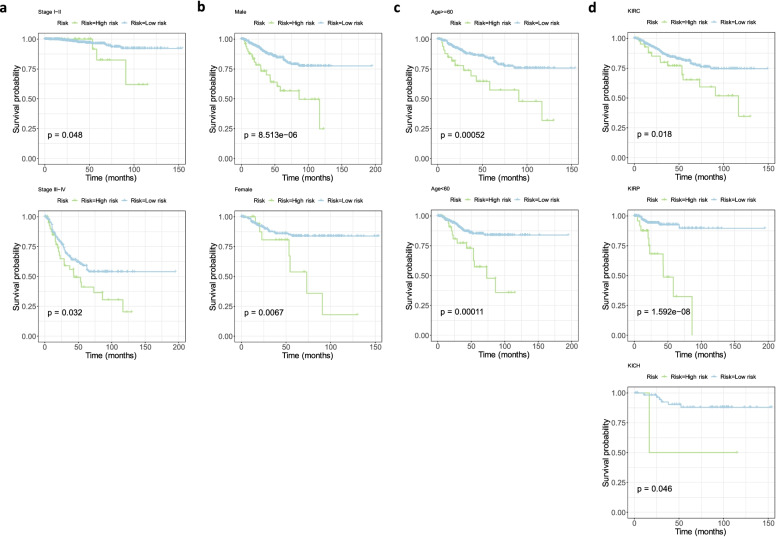
Validating the stability of the prognostic risk model constructed by the 8 selected genes for different subtypes of advanced RCC. Survival plots all showed that high-risk RCC classified by the model resulted in unfavorable OS in different stages (A); genders (B); ages (C) and pathological patterns (C). The p values were shown in the survival plots

The stability of model risk score in different RCC clinical characteristic subgroups of TCGA database was evaluated. There were significant differences between the high and low risk groups according to the age, gender, clinical stage and pathological pattern (Fig. 5A-D). Moreover, it was indicated that the high-risk groups in all subgroups led to adverse prognosis. This showed that the comprehensive prognostic model constructed by the 8genes had good stability.

Besides, we also assessed our model for different end-point events (such as lymph node metastasis or distant metastasis), but the results showed that there was no significant difference between the high-risk and low-risk groups (*P* = 0.18, Supplementary Fig. 2).

### Association of the genes involved in the model with tumor immune infiltrates

**Fig. 6:**
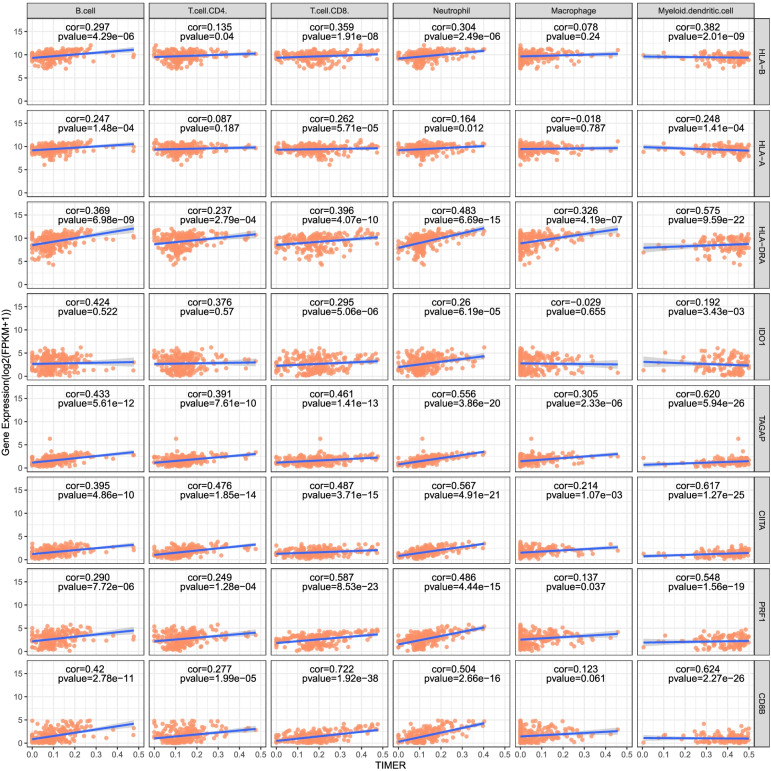
Association of the genes involved in the model with tumor immune infiltrates

The Tumor Immune Assessment Resource (TIMER) platform was used to download the immune score (Supplementary Table 4) of advanced RCC samples. Then we have explored the relationship between the expression of *HLA-B*, *HLA-A*, *HLA-DRA*, *IDO1*, *TAGAP, CIITA*, *PRF1* and *CD8B* at transcriptional level and the tumor infiltrating immune cell populations (B cells, CD8 + T cells, CD4 + , T cells, neutrophils and dendritic cells). We found that the expression levels of *PRF1*, *CIITA*, *TAGAP* and *HLA-DRA* were positively correlated with infiltrates of six types of immune cells in tumors. Additionally, higher infiltration levels of CD8 + T cells, neutrophils and myeloid dendritic cells were significantly correlated with higher expression of the 8 selected genes, respectively (Fig. 6).

## Discussion

The renal parenchyma malignant tumors originate from the renal tubular epithelial cells. Papillary renal cell carcinoma, chromophobe cell renal carcinoma in non-clear-cell renal cell carcinoma and clear cell renal cell carcinoma are three main subtypes of RCC. In recent years, with development of genetic chip and high-throughput sequencing technology, Researches on pathogenesis, prognosis and treatment for RCC have made considerable progress [20]. Our research is of importance for the research field. RCC is regarded as a tumor in a pre-activated immune state and is believed to have a better response to immunotherapy. Mining tumor immune-related biomarkers will help to find out potential targets for the diagnosis and treatment of RCC and will serve as predictors for disease progression, which is prerequisite for quality of life improvement and long-term survival of patients with RCC.

In the present article, we discuss the role of four previously described immune-related gene signatures [21, 22], namely the IFN gamma, the expanded immune gene, the CTL signature, and the HLA-A and HLA-B molecules, in the prognosis of the early stage RCC (stages I and II) and the advanced stage group (stages III and IV). We found that each of the four signatures established a prediction model dividing RCC samples into high- and low-risk groups. Especially in the advanced RCC samples, the high-risk group had significantly worse OS than the low-risk group. Thereafter, we chose 8 genes, *HLA-B*, *HLA-A*, *HLA-DRA*, *IDO1*, *TAGAP*, *CIITA*, *PRF1* and *CD8B*, from the four signatures which were most likely to be related to CSS in advanced RCC. These genes were combined to construct a comprehensive risk model to assess the CSS of the advanced RCC. It similarly implied an unfavorable CSS prognosis in high-risk group of advanced RCC. In a further step, we used external datasets, 91 RCC samples from ICGC database, to verify satisfactory stability of the model.

The 8 selected immune-related genes in this combination played pivotal roles in different biological processes of the tumor growth such as proliferation, apoptosis, metastasis and metabolism. There were many subtypes in the human leukocyte antigen (HLA) system participating in human immune response. According to the structure and function, the HLA genes are divided into two classes, class I and II. Goebel et al. found that the frequency of HLA subtypes impacts RCC development [23]. It was indicated that the high expression of HLA-A and HLA-B which belong to class I in the ccRCC showed better prognosis than those with low expression [24]. HLA-DRA is one of the HLA class II alpha chain paralogues [23]. And Class II transactivator (CIITA) is one of the HLA class II regulatory genes playing a role in inducing the expression of other immune system genes. Butler and Blanck suggested that the expression of the two HLA class II molecules had high level correlation with pRCC [25]. Indoleamine 2,3-Dioxygenase 1 (IDO1) is a tryptophan catabolic enzyme that modifies inflammation and promotes cancer. IDO inhibitors can be used as immune-metabolic adjuvants which safely and potently facilitate the efficacy of immunotherapy [26]. T-cell activation Rho GTPase-activating protein (TAGAP) is a GAP-domain containing protein and was found to exert a role in T-cell differentiation [27]. ZHAO et al. reported that the expression level of TAGAP was related to the positive number of lymph nodes in the prostate cancer [27]. Perforin 1 (PRF1) encodes a protein with structural similarities to complement component C9 that is important in immunity. The protein can form membrane pores releasing granzymes, thus leading to the cytolysis of the target cells [28]. CD8B and CD8A are heterodimers of CD8 (a glycoprotein) expressed only on those cytotoxic T cells to regulate maturation of T cells. Lee found that CD8B gene expression was closely correlated with tumor-infiltrating lymphocytes (TILs) in breast cancer [29]. All mentioned above could be the reasons that the novel prognostic risk model composed of 8 selected immune-related genes could divide the advanced RCC samples into low risk and high risk groups with significant difference in CSS based on the cutoff value. Whether the cutoff value could become anther evaluation index to improve efficacy of IMDC risk classification, which needs further verification.

We have constructed an integrative prediction model with 8 above genes which was able to divide the advanced RCC samples into high-risk and low-risk groups clearly and precisely. Notably, the predictive stability of the model was verified not only by analysis of external data from ICGC database, but also in the subtypes of the samples, including age, gender and clinical stage and pathological pattern. Afterwards, we analyzed the gene mutations in the high-risk and low-risk groups of the advanced kidney cancer and discovered the mutation of some genes, such as *VHL*, *CHEK2, BAP1, PBRM1, which* were closely related to RCC [30–32]. CcRCC and nccRCC are heterogeneous cancers with different histologic, molecular, and genetic alterations. ccRCC, the most common subtype, is strongly associated with the mutation of von Hippel-Lindau (VHL). Targeted therapies for VHL and its related molecular, such as vascular endothelial growth factor (VEGF), platelet-derived growth factor (PDGF) and mammalian target of rapamycin (mTOR), are currently extensively studied [33]. Though most of genetic mutations in ccRCC can be found in nccRCC, we can still detect some characteristic differences in the subtypes of nccRCC. It was reported that abnormal mutations of the MET proto-oncogene and gene encoding fumarate hydratase occurred in Type I and Type II pRCC, respectively [34]. The mutations of TP53 and PTEN could be detected in chRCC [35]. These mutant genes may be potential therapeutic targets for nccRCC. Notably, we found the mutant ratio of ATRX was significantly higher in the high-risk group than that in the low-risk group. The ATRX protein is a chromatin remodeling factor functioning as a transcriptional regulator [36]. The mutation of ATRX was found in various cancers [37]. We hypothesized that the different mutation frequency of the genes may result in different prognosis in high-risk and low-risk groups.

From the above, the 8 selected genes were not only related to immune response, but also in connection with tumors. It suggested that the genes involved in immune activation might affect development of RCC. Therefore, we further investigated the connection between the composing genes and the tumor infiltrating cells via TIMER platform. It is implied that the high expression of the genes favors the immune cells to infiltrate into the tumors. For example, the levels of CD8^+^ T cells, neutrophils and myeloid dendritic cells positively correlate with the expression levels of all selected genes. CD8^+^ T cells, which are a subtype of the cytotoxic T lymphocytes, contribute a lot to the antitumor activity through releasing of tumor cytokines such as INF-γ, perforin and granzyme B [38]. In recent years, studies have confirmed that the tumor-related neutrophils can differentiate into neutrophil type 1 (N1) and neutrophil type 2 (N2) under the influence of the tumor microenvironment. For example, N1 induced by IFN-β functions as anti-tumor neutrophil. In contrast, neutrophils are more likely to become tumor-promoting N2 when the TGF-β pathway is activated [39, 40]. Dendritic cells function as antigen presenting cells and are necessary for the initiation and maintenance of an effective immune response against cancer cells [41]. We suspect that the close relationship between the reported here eight genes and various tumor infiltrating immune cells may be a reason for better prediction of a risk model for advanced RCC development and progression.

Normally, the study faces some limitations. First, TCGA database is short of clinical data about the time of patients enrolled or relapsed, the time free interval and the therapeutic regimen for the patients which can influence the OS of each patient. It may result in a decrease in the predictive power of the model. Second, the selection of genes in this study was from previously described immune-related genetic signatures, therefore we may have missed genes with a predictive role which are not included in the studied signatures. Moreover, we noted that it is an in silico analysis without any further experimental verification. Therefore, independent prospective clinical studies to confirm the capacity of the comprehensive prognostic risk model and research on the mutant genes leading to changes in molecular mechanism of the kidney cancer are further needed.

## Conclusion

In conclusion, the prognostic risk models composed of genes selected from four immune-related genetic signatures demonstrated the potential to predict the survival prognosis of patients with advanced RCC, and have certain reference values for the prognosis assessment of the disease. The close relationship between the genes and tumor-infiltrating immune cells helps to provide new directions for immunotherapy to suppress tumor immune escape, and to develop personalized therapeutic regimen for high-risk group of advanced RCC.

## Supplementary Information

**Additional file 1: Supplementary Fig. 1**. 

**Additional file 2: Supplementary Fig. 2.**  The survival plot showed CSS of high-risk and low-risk groups in metastatic RCC.

**Additional file 3: Supplementary Table 1.** The 8 selected genes to construct prognostic risk model and their weight coefficient in the model.

**Additional file 4: Supplementary Table 2. **The clinical outcomes and risk score of each advanced RCC sample from TCGA database calculated by the prognostic risk model with genes combination (TCGA: The Cancer Genome Atlas; RCC: renal cell carcinoma; OS: overall survival). 

**Additional file 5: Supplementary Table 3. **The risk score and group of each advanced RCC sample from ICGC database calculated by the prognostic risk model with genes combination (ICGC: International Cancer Genome Consortium). 

**Additional file 6:** **Supplementary Table 4.** Immune score of each advanced renal cell carcinoma downloaded from TIMER platform (TIMER: The Tumor Immune Assessment Resource TCGA: The Cancer Genome Atlas).

**Additional file 7: Supplementary Table 5. **_landscape_high risk group. 

## Data Availability

The data and information downloaded and analyzed during the present study are available in the UCSC Xena, http://xenabrowser.net/datapages/, International Cancer Genome Consortium, http://icgc.org/, and Tumor Immune Estimation Resource, http://timer.cistrome.org/.

## Abbreviations

RCC: Renal cell carcinoma
TCGA: The Cancer Genome Atlas
ICGC: International Cancer Genome Consortium
KM: Kaplan-Meier
TIMER: Tumor Immune Estimation Resource
OS: Overall survival
DFS: Disease free survival
CSS: Cancer-specific survival
ccRCC: Clear cell renal cell carcinoma
pRCC: Papillary renal cell carcinoma
ccRC: Chromophobe cell renal carcinoma
irAEs: Immune related adverse events
KIRC: Kidney renal clear cell carcinoma
KIRP: Kidney renal papillary cell carcinoma
KICH: Kidney chromophobe
CTL: Cytotoxic T lymphocyte
AUC: Area under curve
N1: Neutrophil type 1
N2: Neutrophil type 2
