# Supplementary material for: A promising Prognostic risk model for advanced renal cell carcinoma (RCC) with immune-related genes
Source: BMC Cancer. 2022 Jun 23;22:691. doi: 10.1186/s12885-022-09755-2 (PMC9229885; doi:10.1186/s12885-022-09755-2)

**a**

Early Renal cell carcinoma

| Group | Gene | DFS HR | DFS Low 95%CI | DFS High 95%CI | DFS P value | OS HR | OS Low 95%CI | OS High 95%CI | OS P value |
|---|---|---|---|---|---|---|---|---|---|
| HLA | HLA-A | 1.272 | 0.923 | 1.753 | 0.142 | 0.91 | 0.542 | 1.53 | 0.723 |
| | HLA-B | 1.075 | 0.791 | 1.46 | 0.644 | 0.833 | 0.488 | 1.424 | 0.504 |
| IFN-gamma signature | IDO1 | 1.106 | 0.937 | 1.307 | 0.234 | 1.385 | 1.024 | 1.874 | 0.035 |
| | CXCL10 | 1.01 | 0.879 | 1.161 | 0.884 | 1.131 | 0.88 | 1.455 | 0.336 |
| | CXCL9 | 1.087 | 0.952 | 1.241 | 0.216 | 1.172 | 0.926 | 1.482 | 0.186 |
| | HLA-DRA | 1.131 | 0.94 | 1.361 | 0.192 | 1.14 | 0.816 | 1.594 | 0.443 |
| | IFNG | 1.283 | 0.805 | 2.046 | 0.294 | 1.215 | 0.52 | 2.838 | 0.653 |
| Extended immune gene signature | TNFRSF8 | 1.264 | 0.382 | 4.179 | 0.701 | 2.57 | 0.509 | 12.98 | 0.253 |
| | CIITA | 1.159 | 0.825 | 1.629 | 0.394 | 1.468 | 0.815 | 2.644 | 0.201 |
| | CD3E | 1.19 | 0.981 | 1.445 | 0.078 | 1.236 | 0.873 | 1.749 | 0.232 |
| | CCL5 | 1.154 | 0.976 | 1.363 | 0.093 | 1.213 | 0.899 | 1.636 | 0.206 |
| | GZMK | 1.104 | 0.937 | 1.302 | 0.238 | 1.102 | 0.82 | 1.48 | 0.521 |
| | CD2 | 1.162 | 0.971 | 1.39 | 0.102 | 1.21 | 0.875 | 1.673 | 0.249 |
| | CXCL13 | 1.296 | 1.074 | 1.563 | 0.007 | 1.494 | 1.1 | 2.03 | 0.01 |
| | NKG7 | 1.094 | 0.935 | 1.28 | 0.261 | 1.116 | 0.843 | 1.477 | 0.444 |
| | HLA-E | 1.026 | 0.77 | 1.367 | 0.86 | 0.887 | 0.536 | 1.467 | 0.641 |
| | CXCR6 | 1.273 | 0.892 | 1.816 | 0.184 | 1.429 | 0.758 | 2.693 | 0.27 |
| | LAG3 | 1.145 | 0.912 | 1.439 | 0.244 | 1.204 | 0.815 | 1.779 | 0.352 |
| | TAGAP | 0.904 | 0.657 | 1.245 | 0.537 | 0.837 | 0.462 | 1.518 | 0.558 |
| | STAT1 | 1.331 | 1.051 | 1.686 | 0.018 | 1.285 | 0.831 | 1.986 | 0.26 |
| | GZMB | 1.234 | 0.96 | 1.586 | 0.101 | 1.582 | 1.026 | 2.438 | 0.038 |
| Cytotoxic T lymphocyte signature | CD8A | 1.14 | 0.966 | 1.345 | 0.122 | 1.154 | 0.861 | 1.548 | 0.337 |
| | CD8B | 1.164 | 0.944 | 1.437 | 0.156 | 1.142 | 0.782 | 1.669 | 0.491 |
| | GZMA | 1.079 | 0.905 | 1.286 | 0.395 | 1.168 | 0.855 | 1.597 | 0.33 |
| | PRF1 | 1.079 | 0.877 | 1.326 | 0.473 | 1.179 | 0.818 | 1.7 | 0.377 |

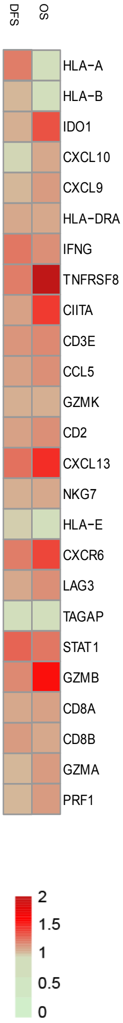


**b**

Advanced Renal cell carcinoma

| Group | Gene | DFS HR | DFS Low 95%CI | DFS High 95%CI | DFS P value | OS HR | OS Low 95%CI | OS High 95%CI | OS P value |
|---|---|---|---|---|---|---|---|---|---|
| HLA | HLA-A | 1.019 | 0.83 | 1.251 | 0.86 | 0.985 | 0.767 | 1.266 | 0.907 |
| | HLA-B | 0.955 | 0.786 | 1.161 | 0.647 | 0.853 | 0.68 | 1.07 | 0.169 |
| IFN-gamma signature | IDO1 | 1.024 | 0.911 | 1.151 | 0.688 | 0.934 | 0.811 | 1.076 | 0.344 |
| | CXCL10 | 1.021 | 0.925 | 1.127 | 0.685 | 1.001 | 0.888 | 1.129 | 0.982 |
| | CXCL9 | 1.026 | 0.936 | 1.124 | 0.586 | 0.984 | 0.881 | 1.098 | 0.77 |
| | HLA-DRA | 0.969 | 0.867 | 1.083 | 0.576 | 0.881 | 0.774 | 1.003 | 0.056 |
| | IFNG | 1.07 | 0.826 | 1.387 | 0.609 | 1.133 | 0.844 | 1.522 | 0.405 |
| Extended immune gene signature | TNFRSF8 | 1.961 | 1.145 | 3.361 | 0.014 | 2.546 | 1.38 | 4.698 | 0.003 |
| | CIITA | 0.898 | 0.716 | 1.125 | 0.349 | 0.837 | 0.636 | 1.101 | 0.204 |
| | CD3E | 1.035 | 0.918 | 1.167 | 0.577 | 0.996 | 0.86 | 1.154 | 0.96 |
| | CCL5 | 1.075 | 0.969 | 1.193 | 0.172 | 1.056 | 0.93 | 1.199 | 0.404 |
| | GZMK | 1.005 | 0.906 | 1.114 | 0.926 | 0.978 | 0.862 | 1.11 | 0.734 |
| | CD2 | 1.03 | 0.922 | 1.15 | 0.601 | 0.984 | 0.859 | 1.127 | 0.814 |
| | CXCL13 | 1.156 | 1.037 | 1.287 | 0.009 | 1.177 | 1.041 | 1.331 | 0.009 |
| | NKG7 | 1.053 | 0.951 | 1.167 | 0.318 | 1.014 | 0.895 | 1.15 | 0.827 |
| | HLA-E | 0.975 | 0.803 | 1.184 | 0.796 | 0.856 | 0.682 | 1.076 | 0.182 |
| | CXCR6 | 1.027 | 0.832 | 1.267 | 0.807 | 1.028 | 0.795 | 1.329 | 0.832 |
| | LAG3 | 1.078 | 0.942 | 1.234 | 0.272 | 1.088 | 0.927 | 1.276 | 0.302 |
| | TAGAP | 0.821 | 0.656 | 1.028 | 0.086 | 0.812 | 0.617 | 1.069 | 0.137 |
| | STAT1 | 1.092 | 0.927 | 1.285 | 0.292 | 1.075 | 0.881 | 1.311 | 0.475 |
| | GZMB | 1.249 | 1.047 | 1.49 | 0.014 | 1.172 | 0.956 | 1.437 | 0.126 |
| Cytotoxic T lymphocyte signature | CD8A | 1.014 | 0.911 | 1.129 | 0.801 | 0.988 | 0.867 | 1.125 | 0.851 |
| | CD8B | 1.008 | 0.877 | 1.159 | 0.906 | 0.977 | 0.825 | 1.156 | 0.784 |
| | GZMA | 1.03 | 0.925 | 1.148 | 0.59 | 0.986 | 0.863 | 1.125 | 0.83 |
| | PRF1 | 1.022 | 0.889 | 1.175 | 0.756 | 0.951 | 0.801 | 1.128 | 0.561 |

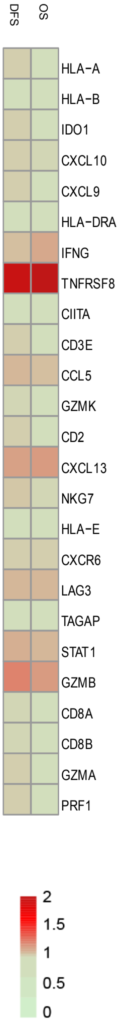

Supplement: Supplementary file 1 — Additional file 1: Supplementary Fig. 1. [file 12885_2022_9755_MOESM1_ESM.pdf]
