# Supplementary material for: A promising Prognostic risk model for advanced renal cell carcinoma (RCC) with immune-related genes
Source: BMC Cancer. 2022 Jun 23;22:691. doi: 10.1186/s12885-022-09755-2 (PMC9229885; doi:10.1186/s12885-022-09755-2)

Supplementary figure 2: The survival plot showed CSS of high-risk and low-risk groups in metastatic RCC.

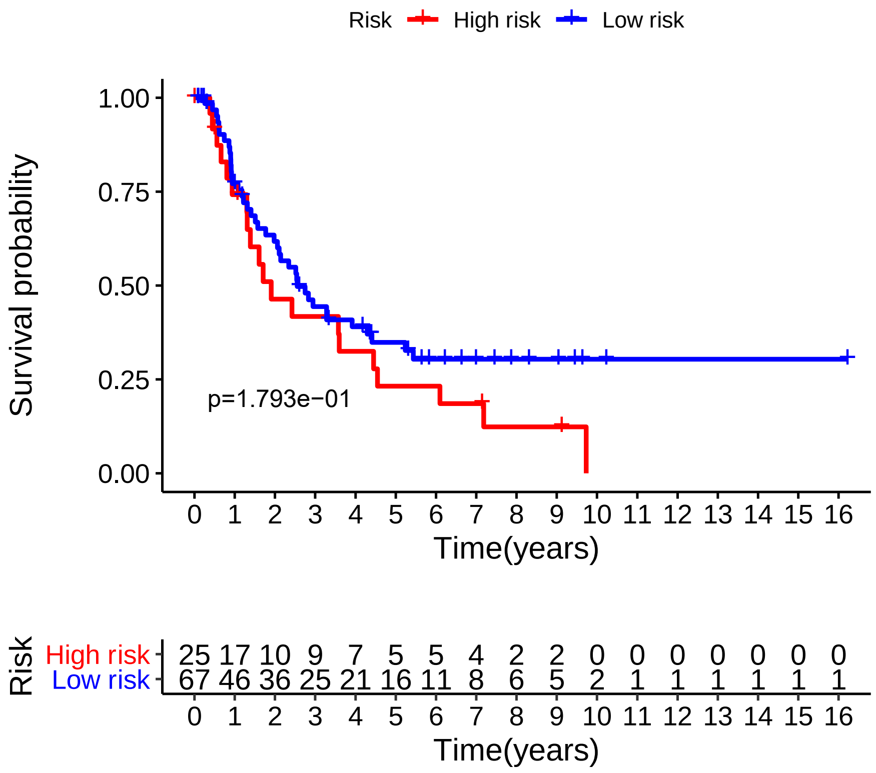

Supplement: Supplementary file 2 — Additional file 2: Supplementary Fig. 2. The survival plot showed CSS of high-risk and low-risk groups in metastatic RCC. [file 12885_2022_9755_MOESM2_ESM.pdf]
