# Supplementary material for: A promising Prognostic risk model for advanced renal cell carcinoma (RCC) with immune-related genes
Source: BMC Cancer. 2022 Jun 23;22:691. doi: 10.1186/s12885-022-09755-2 (PMC9229885; doi:10.1186/s12885-022-09755-2)
Supplement: Supplementary file 3 — Additional file 3: Supplementary Table 1. The 8 selected genes to construct prognostic risk model and their weight coefficient in the model. [file 12885_2022_9755_MOESM3_ESM.docx]

| Gene | Weight coefficient |
| --- | --- |
| HLA-B | -0.60635 |
| HLA-A | 0.367321 |
| HLA-DRA | -0.11771 |
| IDO1 | 0.258753 |
| TAGAP | -0.21705 |
| CIITA | -0.0049 |
| PRF1 | -0.05969 |
| CD8B | 0.39387 |

Supplementary Table 1. The 8 selected genes to construct prognostic risk model and their weight coefficient in the model.
