# Supplementary material for: A promising Prognostic risk model for advanced renal cell carcinoma (RCC) with immune-related genes
Source: BMC Cancer. 2022 Jun 23;22:691. doi: 10.1186/s12885-022-09755-2 (PMC9229885; doi:10.1186/s12885-022-09755-2)
Supplement: Supplementary file 4 — Additional file 4: Supplementary Table 2. The clinical outcomes and risk score of each advanced RCC sample from TCGA database calculated by the prognostic risk model with genes combination (TCGA: The Cancer Genome Atlas; RCC: renal cell carcinoma; OS: overall survival). [file 12885_2022_9755_MOESM4_ESM.docx]

Supplementary Table 2. The clinical outcomes and risk score of each advanced RCC sample from TCGA database calculated by the prognostic risk model with genes combination (TCGA: The Cancer Genome Atlas; RCC: renal cell carcinoma; OS: overall survival)

| Sample | OS(month) | Age(year) | Gender | Stage | Risk score | Risk |
| --- | --- | --- | --- | --- | --- | --- |
| TCGA-2Z-A9JI-01A | 51.48 | 38 | Male | Stage III | -2.76706 | Low risk |
| TCGA-2Z-A9JK-01A | 38.96 | 50 | Male | Stage III | -3.13704 | Low risk |
| TCGA-5P-A9JU-01A | 12.25 | 81 | Male | Stage III | -2.2346 | Low risk |
| TCGA-A3-3307-01A | 47.17 | 66 | Male | Stage III | -2.0462 | High risk |
| TCGA-A3-3308-01A | 0.53 | 77 | Female | Stage III | -2.75701 | Low risk |
| TCGA-A3-3347-01A | 52.89 | 76 | Female | Stage III | -3.30454 | Low risk |
| TCGA-A3-3372-01A | 24.15 | 64 | Male | Stage III | -2.55176 | Low risk |
| TCGA-A3-A8OW-01A | 10.61 | 37 | Male | Stage III | -3.3202 | Low risk |
| TCGA-A4-8310-01A | 25.62 | 58 | Male | Stage III | -3.3272 | Low risk |
| TCGA-A4-8516-01A | 0.76 | 62 | Male | Stage III | -3.49835 | Low risk |
| TCGA-A4-A5Y1-01A | 12.98 | 75 | Male | Stage III | -3.71682 | Low risk |
| TCGA-A4-A7UZ-01A | 0 | 71 | Male | Stage III | -2.03228 | High risk |
| TCGA-AK-3426-01A | 29.07 | 37 | Male | Stage III | -1.64781 | High risk |
| TCGA-AK-3428-01A | 122.47 | 62 | Male | Stage III | -2.09426 | High risk |
| TCGA-AK-3445-01A | 78.58 | 69 | Male | Stage III | -3.75179 | Low risk |
| TCGA-B0-4696-01A | 28.45 | 58 | Male | Stage III | -2.94876 | Low risk |
| TCGA-B0-4710-01A | 57.65 | 75 | Female | Stage III | -2.20465 | Low risk |
| TCGA-B0-4718-01A | 58.41 | 57 | Male | Stage III | -1.77371 | High risk |
| TCGA-B0-4810-01A | 15.7 | 47 | Male | Stage III | -2.10556 | High risk |
| TCGA-B0-4811-01A | 46.55 | 48 | Male | Stage III | -2.58526 | Low risk |
| TCGA-B0-4827-01A | 29.07 | 77 | Female | Stage III | -2.76994 | Low risk |
| TCGA-B0-4842-01A | 56.64 | 73 | Female | Stage III | -2.84367 | Low risk |
| TCGA-B0-4848-01A | 29.01 | 54 | Male | Stage III | -2.56726 | Low risk |
| TCGA-B0-4849-01A | 2.27 | 51 | Male | Stage III | -2.97739 | Low risk |
| TCGA-B0-5081-01A | 11.89 | 79 | Female | Stage III | -2.71163 | Low risk |
| TCGA-B0-5097-01A | 21.85 | 59 | Female | Stage III | -2.74172 | Low risk |
| TCGA-B0-5100-01A | 62.84 | 72 | Male | Stage III | -2.58037 | Low risk |
| TCGA-B0-5108-01A | 58.54 | 54 | Male | Stage III | -2.77498 | Low risk |
| TCGA-B0-5109-01A | 19.28 | 69 | Male | Stage III | -2.00228 | High risk |
| TCGA-B0-5113-01A | 38.6 | 69 | Female | Stage III | -3.07848 | Low risk |
| TCGA-B0-5116-01A | 41.85 | 52 | Male | Stage III | -2.52853 | Low risk |
| TCGA-B0-5400-01A | 56.93 | 59 | Female | Stage III | -2.83923 | Low risk |
| TCGA-B0-5692-01A | 129.57 | 66 | Female | Stage III | -1.98618 | High risk |
| TCGA-B0-5694-01A | 15.77 | 71 | Male | Stage III | -2.62935 | Low risk |
| TCGA-B0-5696-01A | 85.71 | 69 | Male | Stage III | -2.10936 | High risk |
| TCGA-B0-5701-01A | 80.85 | 65 | Male | Stage III | -2.3952 | Low risk |
| TCGA-B0-5709-01A | 130.55 | 62 | Female | Stage III | -2.76144 | Low risk |
| TCGA-B0-5711-01A | 131.04 | 50 | Male | Stage III | -2.77981 | Low risk |
| TCGA-B0-5713-01A | 91.39 | 75 | Female | Stage III | -2.74785 | Low risk |
| TCGA-B1-5398-01A | 41.1 | 73 | Male | Stage III | -3.30655 | Low risk |
| TCGA-B1-A47M-01A | 21.65 | 79 | Male | Stage III | -3.26301 | Low risk |
| TCGA-B3-3925-01A | 33.8 | 52 | Male | Stage III | -3.45372 | Low risk |
| TCGA-B4-5832-01A | 5.09 | 65 | Male | Stage III | -3.36021 | Low risk |
| TCGA-B8-4151-01A | 42.67 | 51 | Female | Stage III | -2.18284 | High risk |
| TCGA-B8-4620-01A | 25.53 | 70 | Female | Stage III | -3.30922 | Low risk |
| TCGA-B8-5158-01A | 40.01 | 56 | Male | Stage III | -2.28711 | Low risk |
| TCGA-B8-5163-01A | 27 | 63 | Female | Stage III | -2.83255 | Low risk |
| TCGA-B8-5164-01A | 0.85 | 65 | Male | Stage III | -2.50679 | Low risk |
| TCGA-B8-5550-01A | 48.49 | 71 | Male | Stage III | -3.01289 | Low risk |
| TCGA-B8-A54D-01A | 27.27 | 69 | Male | Stage III | -2.52268 | Low risk |
| TCGA-B9-5155-01A | 21.85 | 71 | Male | Stage III | -2.91072 | Low risk |
| TCGA-B9-A44B-01A | 11.86 | 70 | Male | Stage III | -1.94089 | High risk |
| TCGA-B9-A69E-01A | 14.85 | 71 | Male | Stage III | -3.00988 | Low risk |
| TCGA-BP-4160-01A | 94.65 | 67 | Male | Stage III | -2.03806 | High risk |
| TCGA-BP-4163-01A | 93.27 | 60 | Female | Stage III | -2.91744 | Low risk |
| TCGA-BP-4166-01A | 0.43 | 69 | Male | Stage III | -2.95702 | Low risk |
| TCGA-BP-4167-01A | 89.29 | 59 | Male | Stage III | -2.42979 | Low risk |
| TCGA-BP-4329-01A | 27.76 | 75 | Male | Stage III | -2.70982 | Low risk |
| TCGA-BP-4330-01A | 62.02 | 60 | Female | Stage III | -2.42854 | Low risk |
| TCGA-BP-4332-01A | 37.22 | 36 | Male | Stage III | -2.60443 | Low risk |
| TCGA-BP-4334-01A | 21.19 | 56 | Male | Stage III | -3.72721 | Low risk |
| TCGA-BP-4343-01A | 62.81 | 64 | Male | Stage III | -2.91621 | Low risk |
| TCGA-BP-4345-01A | 49.8 | 62 | Male | Stage III | -2.84092 | Low risk |
| TCGA-BP-4347-01A | 44.91 | 74 | Male | Stage III | -3.13879 | Low risk |
| TCGA-BP-4351-01A | 31.87 | 51 | Female | Stage III | -2.53154 | Low risk |
| TCGA-BP-4761-01A | 5.98 | 57 | Male | Stage III | -2.1038 | High risk |
| TCGA-BP-4797-01A | 36.37 | 34 | Male | Stage III | -2.92809 | Low risk |
| TCGA-BP-4799-01A | 37.22 | 70 | Male | Stage III | -2.0879 | High risk |
| TCGA-BP-4803-01A | 6.7 | 79 | Male | Stage III | -2.7495 | Low risk |
| TCGA-BP-4967-01A | 6.73 | 76 | Male | Stage III | -2.77894 | Low risk |
| TCGA-BP-4970-01A | 14.22 | 44 | Male | Stage III | -3.40266 | Low risk |
| TCGA-BP-4971-01A | 48.85 | 40 | Male | Stage III | -3.01376 | Low risk |
| TCGA-BP-4972-01A | 49.34 | 43 | Female | Stage III | -2.73076 | Low risk |
| TCGA-BP-4973-01A | 45.47 | 47 | Male | Stage III | -2.58413 | Low risk |
| TCGA-BP-4983-01A | 46.42 | 67 | Female | Stage III | -2.42997 | Low risk |
| TCGA-BP-4985-01A | 31.27 | 72 | Male | Stage III | -3.33575 | Low risk |
| TCGA-BP-4989-01A | 3.88 | 58 | Male | Stage III | -2.04303 | High risk |
| TCGA-BP-5010-01A | 28.84 | 63 | Male | Stage III | -2.1507 | High risk |
| TCGA-BP-5183-01A | 42.41 | 57 | Male | Stage III | -2.80879 | Low risk |
| TCGA-BP-5191-01A | 31.77 | 79 | Male | Stage III | -2.2886 | Low risk |
| TCGA-BP-5198-01A | 19.81 | 72 | Male | Stage III | -3.04696 | Low risk |
| TCGA-BP-5202-01A | 0.95 | 75 | Male | Stage III | -3.31734 | Low risk |
| TCGA-BQ-5875-01A | 86.7 | 66 | Female | Stage III | -2.56071 | Low risk |
| TCGA-BQ-5878-01A | 63.67 | 79 | Female | Stage III | -2.66859 | Low risk |
| TCGA-BQ-5879-01A | 22.9 | 32 | Female | Stage III | -2.14282 | High risk |
| TCGA-BQ-5880-01A | 43.17 | 79 | Male | Stage III | -2.06887 | High risk |
| TCGA-BQ-5882-01A | 21.62 | 54 | Male | Stage III | -2.1533 | High risk |
| TCGA-BQ-5885-01A | 30.26 | 63 | Male | Stage III | -1.76389 | High risk |
| TCGA-BQ-5886-01A | 26.02 | 75 | Male | Stage III | -2.97798 | Low risk |
| TCGA-BQ-5887-01A | 25.33 | 54 | Male | Stage III | -2.88923 | Low risk |
| TCGA-BQ-5890-01A | 7.65 | 69 | Male | Stage III | -2.45885 | Low risk |
| TCGA-BQ-5891-01A | 0.95 | 57 | Female | Stage III | -3.42205 | Low risk |
| TCGA-BQ-7044-01A | 123.52 | 73 | Male | Stage III | -3.01354 | Low risk |
| TCGA-BQ-7048-01A | 93.27 | 64 | Male | Stage III | -3.68801 | Low risk |
| TCGA-BQ-7053-01A | 49.8 | 57 | Female | Stage III | -3.55946 | Low risk |
| TCGA-BQ-7056-01A | 20.11 | 75 | Female | Stage III | -2.12652 | High risk |
| TCGA-BQ-7058-01A | 11.27 | 81 | Male | Stage III | -2.59885 | Low risk |
| TCGA-CJ-4636-01A | 63.21 | 51 | Male | Stage III | -2.4488 | Low risk |
| TCGA-CJ-4640-01A | 114.32 | 49 | Male | Stage III | -2.60035 | Low risk |
| TCGA-CJ-4869-01A | 83.9 | 49 | Male | Stage III | -2.42648 | Low risk |
| TCGA-CJ-4870-01A | 49.21 | 58 | Female | Stage III | -3.01208 | Low risk |
| TCGA-CJ-4873-01A | 74.21 | 85 | Female | Stage III | -2.8535 | Low risk |
| TCGA-CJ-4878-01A | 71.81 | 71 | Female | Stage III | -2.46249 | Low risk |
| TCGA-CJ-4881-01A | 66.16 | 41 | Male | Stage III | -2.8772 | Low risk |
| TCGA-CJ-4882-01A | 61.86 | 57 | Male | Stage III | -2.42359 | Low risk |
| TCGA-CJ-4884-01A | 57.79 | 72 | Female | Stage III | -2.67795 | Low risk |
| TCGA-CJ-4894-01A | 27.63 | 58 | Male | Stage III | -2.57545 | Low risk |
| TCGA-CJ-4897-01A | 109.76 | 79 | Female | Stage III | -2.72229 | Low risk |
| TCGA-CJ-4901-01A | 47.63 | 47 | Male | Stage III | -2.08852 | High risk |
| TCGA-CJ-4902-01A | 49.93 | 61 | Male | Stage III | -2.50431 | Low risk |
| TCGA-CJ-4907-01A | 49.24 | 58 | Male | Stage III | -2.75613 | Low risk |
| TCGA-CJ-4916-01A | 45.11 | 69 | Female | Stage III | -1.95086 | High risk |
| TCGA-CJ-5676-01A | 133.61 | 47 | Male | Stage III | -2.49356 | Low risk |
| TCGA-CJ-5679-01A | 22.31 | 73 | Male | Stage III | -3.10554 | Low risk |
| TCGA-CJ-5684-01A | 73.29 | 61 | Male | Stage III | -2.93546 | Low risk |
| TCGA-CW-5584-01A | 5.39 | 74 | Male | Stage III | -2.52868 | Low risk |
| TCGA-CW-5587-01A | 73.13 | 62 | Female | Stage III | -2.79526 | Low risk |
| TCGA-CW-6097-01A | 18.76 | 32 | Male | Stage III | -2.90659 | Low risk |
| TCGA-CZ-4863-01A | 63.34 | 51 | Female | Stage III | -2.61428 | Low risk |
| TCGA-CZ-5457-01A | 90.47 | 62 | Male | Stage III | -2.97639 | Low risk |
| TCGA-CZ-5458-01A | 91.62 | 43 | Male | Stage III | -2.29089 | Low risk |
| TCGA-CZ-5459-01A | 55.29 | 63 | Male | Stage III | -2.3494 | Low risk |
| TCGA-CZ-5466-01A | 22.5 | 67 | Male | Stage III | -2.86952 | Low risk |
| TCGA-CZ-5467-01A | 2.4 | 86 | Female | Stage III | -2.27915 | Low risk |
| TCGA-EU-5907-01A | 4.17 | 81 | Male | Stage III | -3.28423 | Low risk |
| TCGA-F9-A4JJ-01A | 10.68 | 35 | Female | Stage III | -2.3407 | Low risk |
| TCGA-F9-A97G-01A | 0.43 | 79 | Male | Stage III | -3.42286 | Low risk |
| TCGA-G7-6797-01A | 25.13 | 46 | Male | Stage III | -2.95735 | Low risk |
| TCGA-G7-7501-01A | 20.5 | 55 | Female | Stage III | -2.00819 | High risk |
| TCGA-G7-A8LD-01A | 16.89 | 71 | Male | Stage III | -2.85405 | Low risk |
| TCGA-GL-7966-01A | 3.68 | 28 | Female | Stage III | -2.46479 | Low risk |
| TCGA-GL-A59R-01A | 12.42 | 79 | Male | Stage III | -3.40917 | Low risk |
| TCGA-HE-7130-01A | 58.15 | NA | Female | Stage III | -2.64609 | Low risk |
| TCGA-IA-A40U-01A | 16.79 | 56 | Male | Stage III | -3.10574 | Low risk |
| TCGA-IA-A40Y-01A | 2.04 | 60 | Female | Stage III | -3.02146 | Low risk |
| TCGA-J7-8537-01A | 10.55 | 37 | Female | Stage III | -2.50909 | Low risk |
| TCGA-KL-8323-01A | 38.04 | 57 | Female | Stage III | -3.10213 | Low risk |
| TCGA-KL-8326-01A | 109.13 | 69 | Male | Stage III | -3.02536 | Low risk |
| TCGA-KL-8334-01A | 96.88 | 37 | Female | Stage III | -4.05226 | Low risk |
| TCGA-KL-8335-01A | 92.48 | 67 | Male | Stage III | -2.91257 | Low risk |
| TCGA-KL-8338-01A | 86.5 | 51 | Male | Stage III | -3.47614 | Low risk |
| TCGA-KL-8344-01A | 28.78 | 51 | Male | Stage III | -3.234 | Low risk |
| TCGA-KL-8345-01A | 54.7 | 75 | Male | Stage III | -4.30989 | Low risk |
| TCGA-KM-8440-01A | 44.88 | 37 | Male | Stage III | -2.75684 | Low risk |
| TCGA-KN-8429-01A | 87.06 | 38 | Female | Stage III | -2.86224 | Low risk |
| TCGA-KN-8433-01A | 11.2 | 46 | Female | Stage III | -3.34756 | Low risk |
| TCGA-KO-8405-01A | 100.39 | 29 | Male | Stage III | -3.49636 | Low risk |
| TCGA-KO-8408-01A | 16.66 | 47 | Male | Stage III | -2.05673 | High risk |
| TCGA-KO-8416-01A | 90.41 | 41 | Male | Stage III | -3.09673 | Low risk |
| TCGA-P4-A5E6-01A | 99.7 | 67 | Male | Stage III | -2.61066 | Low risk |
| TCGA-P4-A5E8-01A | 42.9 | 57 | Male | Stage III | -1.841 | High risk |
| TCGA-P4-A5EA-01A | 6.6 | 54 | Female | Stage III | -2.38236 | Low risk |
| TCGA-P4-AAVK-01A | 52.23 | 74 | Male | Stage III | -3.60432 | Low risk |
| TCGA-P4-AAVL-01A | 16 | 85 | Male | Stage III | -3.20801 | Low risk |
| TCGA-Q2-A5QZ-01A | 14.06 | 61 | Female | Stage III | -3.29076 | Low risk |
| TCGA-UZ-A9PN-01A | 37.32 | 54 | Male | Stage III | -2.53249 | Low risk |
| TCGA-UZ-A9PQ-01A | 86.2 | 59 | Male | Stage III | -2.12922 | High risk |
| TCGA-UZ-A9PZ-01A | 21.58 | 65 | Male | Stage III | -3.01992 | Low risk |
| TCGA-Y8-A896-01A | 18.63 | 62 | Male | Stage III | -3.43334 | Low risk |
| TCGA-2Z-A9J7-01A | 4.57 | 63 | Male | Stage IV | -2.09275 | High risk |
| TCGA-4A-A93X-01A | 12.81 | 58 | Male | Stage IV | -2.15019 | High risk |
| TCGA-AK-3436-01A | 109.43 | 40 | Male | Stage IV | -2.19165 | High risk |
| TCGA-AL-3466-01A | 9.63 | 41 | Male | Stage IV | -1.93525 | High risk |
| TCGA-AL-7173-01A | 67.67 | 72 | Female | Stage IV | -2.55981 | Low risk |
| TCGA-B0-4841-01A | 6.7 | 63 | Male | Stage IV | -1.59618 | High risk |
| TCGA-B0-4844-01A | 10.28 | 60 | Male | Stage IV | -3.09067 | Low risk |
| TCGA-B0-4845-01A | 65.24 | 70 | Male | Stage IV | -2.54421 | Low risk |
| TCGA-B0-4846-01A | 39.42 | 52 | Male | Stage IV | -2.99939 | Low risk |
| TCGA-B0-5080-01A | 11.24 | 63 | Male | Stage IV | -2.15006 | High risk |
| TCGA-B0-5084-01A | 7.29 | 33 | Male | Stage IV | -2.70145 | Low risk |
| TCGA-B0-5094-01A | 10.94 | 62 | Male | Stage IV | -3.0619 | Low risk |
| TCGA-B0-5107-01A | 30.45 | 65 | Female | Stage IV | -2.49468 | Low risk |
| TCGA-B0-5115-01A | 52.69 | 43 | Male | Stage IV | -3.24218 | Low risk |
| TCGA-B0-5402-01A | 42.38 | 64 | Male | Stage IV | -2.54251 | Low risk |
| TCGA-B0-5712-01A | 89.42 | 68 | Female | Stage IV | -2.67671 | Low risk |
| TCGA-B2-5639-01A | 32.95 | 46 | Male | Stage IV | -3.26158 | Low risk |
| TCGA-B4-5377-01A | 11.99 | 68 | Female | Stage IV | -2.95285 | Low risk |
| TCGA-B8-4622-01A | 50.1 | 57 | Male | Stage IV | -2.89597 | Low risk |
| TCGA-BP-4335-01A | 15.6 | 65 | Female | Stage IV | -1.85286 | High risk |
| TCGA-BP-4352-01A | 11.3 | 74 | Female | Stage IV | -2.2654 | Low risk |
| TCGA-BP-4354-01A | 33.97 | 40 | Male | Stage IV | -3.34257 | Low risk |
| TCGA-BP-4770-01A | 10.81 | 73 | Female | Stage IV | -2.57777 | Low risk |
| TCGA-BP-4771-01A | 5.32 | 62 | Male | Stage IV | -2.1654 | High risk |
| TCGA-BP-4787-01A | 15.77 | 59 | Female | Stage IV | -3.39454 | Low risk |
| TCGA-BP-4974-01A | 6.93 | 58 | Male | Stage IV | -2.90209 | Low risk |
| TCGA-BP-5178-01A | 62.81 | 71 | Male | Stage IV | -3.26503 | Low risk |
| TCGA-BP-5201-01A | 31.24 | 63 | Male | Stage IV | -2.20787 | Low risk |
| TCGA-BQ-5877-01A | 8.87 | 60 | Male | Stage IV | -3.74393 | Low risk |
| TCGA-BQ-5889-01A | 10.81 | 63 | Male | Stage IV | -2.51135 | Low risk |
| TCGA-BQ-5893-01A | 7.92 | 61 | Male | Stage IV | -1.59465 | High risk |
| TCGA-BQ-5894-01A | 2.76 | 42 | Male | Stage IV | -3.0563 | Low risk |
| TCGA-CJ-4637-01A | 73.16 | 52 | Female | Stage IV | -1.89032 | High risk |
| TCGA-CJ-4638-01A | 14.16 | 46 | Female | Stage IV | -2.71554 | Low risk |
| TCGA-CJ-4641-01A | 54.57 | 55 | Female | Stage IV | -2.12491 | High risk |
| TCGA-CJ-4644-01A | 11.04 | 48 | Female | Stage IV | -2.40169 | Low risk |
| TCGA-CJ-4868-01A | 21.22 | 42 | Male | Stage IV | -2.44844 | Low risk |
| TCGA-CJ-4871-01A | 79.6 | 63 | Male | Stage IV | -2.54432 | Low risk |
| TCGA-CJ-4875-01A | 116.75 | 67 | Male | Stage IV | -1.76907 | High risk |
| TCGA-CJ-4885-01A | 113.37 | 64 | Male | Stage IV | -2.42024 | Low risk |
| TCGA-CJ-4887-01A | 30.62 | 48 | Male | Stage IV | -2.55289 | Low risk |
| TCGA-CJ-4888-01A | 51.48 | 59 | Male | Stage IV | -2.48275 | Low risk |
| TCGA-CJ-4890-01A | 115.6 | 72 | Male | Stage IV | -2.64581 | Low risk |
| TCGA-CJ-4895-01A | 39.42 | 62 | Male | Stage IV | -3.26987 | Low risk |
| TCGA-CJ-4904-01A | 108.48 | 60 | Female | Stage IV | -2.725 | Low risk |
| TCGA-CJ-4918-01A | 3.06 | 64 | Male | Stage IV | -2.42599 | Low risk |
| TCGA-CJ-5677-01A | 25.69 | 54 | Female | Stage IV | -3.51969 | Low risk |
| TCGA-CJ-5678-01A | 18.86 | 62 | Male | Stage IV | -2.66154 | Low risk |
| TCGA-CJ-5680-01A | 25.23 | 65 | Female | Stage IV | -2.72718 | Low risk |
| TCGA-CJ-5681-01A | 18.13 | 44 | Female | Stage IV | -3.1027 | Low risk |
| TCGA-CJ-5682-01A | 122.73 | 60 | Male | Stage IV | -2.57159 | Low risk |
| TCGA-CJ-6028-01A | 53.38 | 58 | Male | Stage IV | -2.02988 | High risk |
| TCGA-CJ-6033-01A | 7.36 | 54 | Female | Stage IV | -2.29647 | Low risk |
| TCGA-CW-5585-01A | 85.71 | 51 | Male | Stage IV | -2.15717 | High risk |
| TCGA-CW-5590-01A | 35.32 | 51 | Male | Stage IV | -2.46451 | Low risk |
| TCGA-CW-5591-01A | 74.61 | 56 | Male | Stage IV | -2.90868 | Low risk |
| TCGA-CZ-4857-01A | 47.04 | 56 | Male | Stage IV | -3.17863 | Low risk |
| TCGA-CZ-5454-01A | 23.72 | 63 | Male | Stage IV | -2.88688 | Low risk |
| TCGA-CZ-5460-01A | 94.38 | 55 | Male | Stage IV | -2.32478 | Low risk |
| TCGA-CZ-5461-01A | 10.84 | 52 | Male | Stage IV | -2.65361 | Low risk |
| TCGA-CZ-5464-01A | 69.91 | 69 | Male | Stage IV | -2.59287 | Low risk |
| TCGA-CZ-5987-01A | 14.62 | 60 | Male | Stage IV | -2.88165 | Low risk |
| TCGA-F9-A8NY-01A | 1.18 | 38 | Female | Stage IV | -2.53322 | Low risk |
| TCGA-G6-A8L6-01A | 10.28 | 55 | Male | Stage IV | -2.55421 | Low risk |
| TCGA-G7-A8LB-01A | 17.97 | 70 | Male | Stage IV | -1.43941 | High risk |
| TCGA-GL-6846-01A | 194.65 | 52 | Male | Stage IV | -2.75793 | Low risk |
| TCGA-KL-8336-01A | 30.22 | 51 | Female | Stage IV | -3.00009 | Low risk |
| TCGA-KL-8339-01A | 28.09 | 67 | Male | Stage IV | -2.45863 | Low risk |
| TCGA-KL-8341-01A | 24.77 | 41 | Male | Stage IV | -2.82819 | Low risk |
| TCGA-KN-8426-01A | 3.55 | 50 | Male | Stage IV | -3.23846 | Low risk |
| TCGA-KN-8427-01A | 0.99 | 54 | Male | Stage IV | -2.39267 | Low risk |
| TCGA-KO-8404-01A | 10.68 | 78 | Male | Stage IV | -2.70849 | Low risk |
