# Supplementary material for: A promising Prognostic risk model for advanced renal cell carcinoma (RCC) with immune-related genes
Source: BMC Cancer. 2022 Jun 23;22:691. doi: 10.1186/s12885-022-09755-2 (PMC9229885; doi:10.1186/s12885-022-09755-2)
Supplement: Supplementary file 5 — Additional file 5: Supplementary Table 3. The risk score and group of each advanced RCC sample from ICGC database calculated by the prognostic risk model with genes combination (ICGC: International Cancer Genome Consortium). [file 12885_2022_9755_MOESM5_ESM.docx]

Supplementary Table 3. The risk score and group of each advanced RCC sample from ICGC database calculated by the prognostic risk model with genes combination (ICGC: International Cancer Genome Consortium)

| Sample | Risk score | Group |
| --- | --- | --- |
| DO46827 | -2.93548 | Low risk |
| DO46828 | -1.89176 | High risk |
| DO46830 | -2.6036 | High risk |
| DO46832 | -1.95463 | High risk |
| DO46834 | -1.91153 | High risk |
| DO46838 | -2.62202 | Low risk |
| DO46841 | -2.71256 | Low risk |
| DO46844 | -2.19237 | High risk |
| DO46847 | -2.48298 | High risk |
| DO46850 | -2.31497 | High risk |
| DO46853 | -1.6918 | High risk |
| DO46856 | -2.81694 | Low risk |
| DO46859 | -2.14337 | High risk |
| DO46862 | -2.84435 | Low risk |
| DO46865 | -1.88806 | High risk |
| DO46873 | -2.42264 | High risk |
| DO46877 | -2.21008 | High risk |
| DO46889 | -2.51815 | High risk |
| DO46893 | -1.91615 | High risk |
| DO46905 | -2.82243 | Low risk |
| DO46909 | -2.2292 | High risk |
| DO46913 | -3.16909 | Low risk |
| DO46917 | -2.90824 | Low risk |
| DO46925 | -2.10751 | High risk |
| DO46933 | -3.22077 | Low risk |
| DO46941 | -2.86883 | Low risk |
| DO46945 | -3.10557 | Low risk |
| DO46949 | -1.82738 | High risk |
| DO46953 | -3.22628 | Low risk |
| DO46957 | -2.6831 | Low risk |
| DO46961 | -2.29662 | High risk |
| DO46965 | -2.71209 | Low risk |
| DO46969 | -2.19491 | High risk |
| DO46973 | -2.42794 | High risk |
| DO46980 | -2.24876 | High risk |
| DO46984 | -2.97346 | Low risk |
| DO46988 | -2.56382 | High risk |
| DO46992 | -2.10989 | High risk |
| DO46996 | -2.66125 | Low risk |
| DO47000 | -2.64934 | Low risk |
| DO47004 | -2.53556 | High risk |
| DO47008 | -2.51681 | High risk |
| DO47012 | -2.35837 | High risk |
| DO47016 | -2.99999 | Low risk |
| DO47020 | -2.54692 | High risk |
| DO47024 | -2.16988 | High risk |
| DO47028 | -2.71906 | Low risk |
| DO47032 | -2.08165 | High risk |
| DO47036 | -2.86228 | Low risk |
| DO47040 | -2.78007 | Low risk |
| DO47044 | -3.49673 | Low risk |
| DO47048 | -2.71133 | Low risk |
| DO47056 | -1.49087 | High risk |
| DO47068 | -2.30877 | High risk |
| DO47072 | -2.35892 | High risk |
| DO47076 | -2.62846 | Low risk |
| DO47080 | -2.93442 | Low risk |
| DO47084 | -2.24809 | High risk |
| DO47096 | -1.86981 | High risk |
| DO47100 | -2.53467 | High risk |
| DO47108 | -3.22122 | Low risk |
| DO47120 | -2.37613 | High risk |
| DO47128 | -2.40781 | High risk |
| DO47132 | -2.01303 | High risk |
| DO47140 | -2.05577 | High risk |
| DO47144 | -2.95664 | Low risk |
| DO47156 | -2.48562 | High risk |
| DO47159 | -2.92016 | Low risk |
| DO47162 | -3.56261 | Low risk |
| DO47168 | -2.49983 | High risk |
| DO47171 | -2.49091 | High risk |
| DO47174 | -2.17809 | High risk |
| DO47177 | -2.75584 | Low risk |
| DO47180 | -2.82943 | Low risk |
| DO47183 | -2.62163 | High risk |
| DO47186 | -2.31473 | High risk |
| DO47189 | -2.57865 | High risk |
| DO47192 | -1.10046 | High risk |
| DO47195 | -2.47546 | High risk |
| DO47198 | -2.21444 | High risk |
| DO47201 | -2.65493 | Low risk |
| DO47204 | -2.86911 | Low risk |
| DO47207 | -2.59513 | High risk |
| DO47210 | -2.45604 | High risk |
| DO47213 | -2.86797 | Low risk |
| DO47216 | -1.94853 | High risk |
| DO47219 | -1.86989 | High risk |
| DO47222 | -2.15903 | High risk |
| DO47225 | -2.64602 | Low risk |
| DO47228 | -3.00303 | Low risk |
| DO47231 | -2.43862 | High risk |
