# Supplementary material for: A promising Prognostic risk model for advanced renal cell carcinoma (RCC) with immune-related genes
Source: BMC Cancer. 2022 Jun 23;22:691. doi: 10.1186/s12885-022-09755-2 (PMC9229885; doi:10.1186/s12885-022-09755-2)
Supplement: Supplementary file 6 — Additional file 6: Supplementary Table 4. Immune score of each advanced renal cell carcinoma downloaded from TIMER platform (TIMER: The Tumor Immune Assessment Resource TCGA: The Cancer Genome Atlas). [file 12885_2022_9755_MOESM6_ESM.docx]

Supplementary Table 4. Immune score of each advanced renal cell carcinoma downloaded from TIMER platform (TIMER: The Tumor Immune Assessment Resource TCGA: The Cancer Genome Atlas)

| **Sample ID** | **B cell** | **CD4^+^ T cell** | **CD8^+^ T cell** | **Neutrophil** | | **Macrophage** | **Myeloid dendritic cell** |
| --- | --- | --- | --- | --- | --- | --- | --- |
| TCGA-2Z-A9J7-01A | 1.28E-02 | 7.65E-02 | 1.54E-01 | 8.08E-02 | 0.00E+00 | | 3.14E-01 |
| TCGA-2Z-A9JI-01A | 2.87E-01 | 1.18E-01 | 2.63E-01 | 1.79E-01 | 1.32E-01 | | 5.94E-01 |
| TCGA-2Z-A9JK-01A | 1.90E-01 | 1.52E-01 | 2.64E-01 | 1.44E-01 | 1.21E-01 | | 5.75E-01 |
| TCGA-4A-A93X-01A | 4.49E-02 | 5.69E-02 | 1.51E-01 | 7.88E-02 | 0.00E+00 | | 3.10E-01 |
| TCGA-5P-A9JU-01A | 7.56E-02 | 9.46E-02 | 1.47E-01 | 8.95E-02 | 0.00E+00 | | 3.66E-01 |
| TCGA-A3-3307-01A | 1.01E-01 | 1.12E-01 | 3.02E-01 | 1.87E-01 | 1.72E-01 | | 5.24E-01 |
| TCGA-A3-3308-01A | 9.51E-02 | 2.33E-01 | 4.74E-02 | 1.16E-01 | 1.47E-01 | | 5.13E-01 |
| TCGA-A3-3347-01A | 1.07E-01 | 3.37E-01 | 1.18E-01 | 3.01E-01 | 4.24E-01 | | 7.41E-01 |
| TCGA-A3-3372-01A | 5.74E-02 | 2.06E-01 | 2.25E-01 | 1.88E-01 | 1.53E-01 | | 4.64E-01 |
| TCGA-A3-A8OW-01A | 7.56E-02 | 2.38E-01 | 1.91E-01 | 1.13E-01 | 2.50E-03 | | 4.66E-01 |
| TCGA-A4-8310-01A | 9.67E-02 | 1.04E-01 | 1.55E-01 | 9.09E-02 | 7.18E-02 | | 4.53E-01 |
| TCGA-A4-8516-01A | 6.75E-02 | 1.07E-01 | 1.86E-01 | 1.09E-01 | 4.86E-02 | | 4.27E-01 |
| TCGA-A4-A5Y1-01A | 1.97E-01 | 6.41E-02 | 2.69E-01 | 9.82E-02 | 1.03E-01 | | 5.28E-01 |
| TCGA-A4-A7UZ-01A | 1.41E-01 | 1.75E-01 | 3.24E-01 | 1.97E-01 | 2.18E-02 | | 5.70E-01 |
| TCGA-AK-3426-01A | 0.00E+00 | 1.78E-01 | 7.70E-01 | 2.66E-01 | 7.06E-02 | | 8.01E-01 |
| TCGA-AK-3428-01A | 7.37E-02 | 0.00E+00 | 1.65E-01 | 1.67E-01 | 5.97E-03 | | 4.30E-01 |
| TCGA-AK-3436-01A | 4.86E-02 | 1.43E-02 | 1.09E-01 | 0.00E+00 | 0.00E+00 | | 1.92E-01 |
| TCGA-AK-3445-01A | 9.37E-02 | 1.78E-01 | 1.78E-01 | 1.08E-01 | 9.48E-02 | | 5.58E-01 |
| TCGA-AL-3466-01A | 1.06E-01 | 4.59E-02 | 2.83E-01 | 1.01E-01 | 3.38E-02 | | 4.57E-01 |
| TCGA-AL-7173-01A | 9.94E-02 | 1.22E-01 | 2.21E-01 | 1.07E-01 | 0.00E+00 | | 4.43E-01 |
| TCGA-B0-4696-01A | 1.76E-01 | 1.21E-01 | 9.69E-02 | 4.34E-02 | 5.36E-02 | | 5.39E-01 |
| TCGA-B0-4710-01A | 6.58E-02 | 1.51E-01 | 1.70E-01 | 1.17E-01 | 0.00E+00 | | 4.29E-01 |
| TCGA-B0-4718-01A | 3.76E-02 | 1.14E-01 | 9.14E-02 | 6.30E-02 | 0.00E+00 | | 2.78E-01 |
| TCGA-B0-4810-01A | 7.16E-02 | 1.91E-01 | 4.67E-01 | 1.87E-01 | 1.89E-02 | | 6.50E-01 |
| TCGA-B0-4811-01A | 0.00E+00 | 6.24E-02 | 0.00E+00 | 0.00E+00 | 0.00E+00 | | 7.57E-02 |
| TCGA-B0-4827-01A | 1.61E-01 | 1.59E-01 | 1.65E-01 | 1.71E-01 | 8.11E-02 | | 5.66E-01 |
| TCGA-B0-4841-01A | 0.00E+00 | 1.88E-02 | 9.81E-02 | 0.00E+00 | 0.00E+00 | | 1.07E-01 |
| TCGA-B0-4842-01A | 1.45E-01 | 2.19E-01 | 3.44E-02 | 1.09E-01 | 0.00E+00 | | 5.19E-01 |
| TCGA-B0-4844-01A | 1.11E-01 | 1.35E-01 | 0.00E+00 | 6.13E-02 | 0.00E+00 | | 2.74E-01 |
| TCGA-B0-4845-01A | 1.78E-02 | 1.99E-01 | 1.54E-02 | 6.17E-02 | 0.00E+00 | | 2.81E-01 |
| TCGA-B0-4846-01A | 2.07E-01 | 2.00E-01 | 1.76E-01 | 2.61E-01 | 4.57E-03 | | 7.16E-01 |
| TCGA-B0-4848-01A | 1.35E-01 | 1.67E-01 | 2.03E-01 | 1.56E-01 | 1.78E-01 | | 6.78E-01 |
| TCGA-B0-4849-01A | 4.17E-02 | 2.06E-01 | 7.50E-02 | 1.54E-01 | 4.85E-02 | | 4.20E-01 |
| TCGA-B0-5080-01A | 2.02E-01 | 3.15E-01 | 2.39E-01 | 1.41E-01 | 9.98E-02 | | 5.83E-01 |
| TCGA-B0-5081-01A | 2.46E-01 | 3.37E-01 | 3.20E-01 | 1.90E-01 | 3.80E-04 | | 7.67E-01 |
| TCGA-B0-5084-01A | 1.75E-02 | 1.40E-01 | 0.00E+00 | 4.80E-02 | 0.00E+00 | | 3.33E-01 |
| TCGA-B0-5094-01A | 7.48E-02 | 7.86E-02 | 1.18E-01 | 6.03E-02 | 0.00E+00 | | 3.37E-01 |
| TCGA-B0-5097-01A | 0.00E+00 | 1.99E-01 | 1.97E-01 | 2.57E-01 | 1.74E-01 | | 6.32E-01 |
| TCGA-B0-5100-01A | 1.85E-02 | 1.64E-01 | 4.04E-02 | 1.63E-02 | 0.00E+00 | | 3.06E-01 |
| TCGA-B0-5107-01A | 1.47E-01 | 1.50E-01 | 1.75E-01 | 6.94E-02 | 0.00E+00 | | 4.99E-01 |
| TCGA-B0-5108-01A | 0.00E+00 | 2.18E-01 | 2.41E-01 | 3.20E-01 | 2.18E-01 | | 7.02E-01 |
| TCGA-B0-5109-01A | 9.49E-02 | 7.93E-02 | 4.73E-01 | 2.83E-01 | 0.00E+00 | | 4.85E-01 |
| TCGA-B0-5113-01A | 1.38E-01 | 1.95E-01 | 1.60E-01 | 1.53E-01 | 5.69E-02 | | 5.60E-01 |
| TCGA-B0-5115-01A | 8.71E-02 | 1.70E-01 | 1.53E-01 | 1.17E-01 | 0.00E+00 | | 4.83E-01 |
| TCGA-B0-5116-01A | 4.22E-02 | 5.66E-02 | 5.63E-02 | 2.21E-02 | 0.00E+00 | | 1.84E-01 |
| TCGA-B0-5400-01A | 3.24E-02 | 1.59E-02 | 1.57E-01 | 1.84E-01 | 0.00E+00 | | 3.58E-01 |
| TCGA-B0-5402-01A | 9.67E-02 | 8.05E-02 | 2.24E-01 | 9.73E-02 | 0.00E+00 | | 3.80E-01 |
| TCGA-B0-5692-01A | 1.44E-01 | 4.52E-05 | 8.63E-01 | 2.04E-01 | 0.00E+00 | | 6.90E-01 |
| TCGA-B0-5694-01A | 5.48E-02 | 5.08E-02 | 1.21E-01 | 7.22E-02 | 0.00E+00 | | 3.54E-01 |
| TCGA-B0-5696-01A | 1.07E-01 | 5.79E-02 | 1.20E-01 | 4.75E-02 | 0.00E+00 | | 3.60E-01 |
| TCGA-B0-5701-01A | 1.50E-01 | 1.04E-01 | 1.30E-01 | 1.19E-01 | 3.69E-02 | | 5.31E-01 |
| TCGA-B0-5709-01A | 1.51E-01 | 4.44E-01 | 2.25E-01 | 3.29E-01 | 2.28E-01 | | 9.05E-01 |
| TCGA-B0-5711-01A | 1.27E-01 | 1.39E-01 | 2.67E-01 | 1.45E-01 | 2.23E-02 | | 5.78E-01 |
| TCGA-B0-5712-01A | 6.57E-02 | 2.64E-02 | 1.42E-01 | 5.05E-02 | 0.00E+00 | | 2.91E-01 |
| TCGA-B0-5713-01A | 5.71E-02 | 1.37E-01 | 2.52E-01 | 1.64E-01 | 0.00E+00 | | 4.32E-01 |
| TCGA-B1-5398-01A | 2.15E-01 | 1.86E-01 | 3.33E-01 | 1.33E-01 | 4.57E-01 | | 7.41E-01 |
| TCGA-B1-A47M-01A | 7.69E-02 | 1.54E-01 | 1.07E-01 | 1.63E-01 | 0.00E+00 | | 4.08E-01 |
| TCGA-B2-5639-01A | 1.26E-01 | 1.31E-01 | 1.15E-01 | 1.58E-01 | 5.14E-02 | | 5.14E-01 |
| TCGA-B3-3925-01A | 1.57E-01 | 1.67E-01 | 2.44E-01 | 1.72E-01 | 2.39E-01 | | 5.69E-01 |
| TCGA-B4-5377-01A | 1.85E-01 | 1.58E-01 | 1.30E-01 | 1.81E-01 | 5.03E-02 | | 5.83E-01 |
| TCGA-B4-5832-01A | 1.61E-01 | 2.18E-01 | 3.38E-01 | 2.27E-01 | 8.91E-01 | | 8.29E-01 |
| TCGA-B8-4151-01A | 1.03E-01 | 3.95E-02 | 3.37E-02 | 3.87E-02 | 0.00E+00 | | 2.93E-01 |
| TCGA-B8-4620-01A | 0.00E+00 | 1.22E-01 | 1.52E-02 | 2.11E-01 | 3.67E-02 | | 4.89E-01 |
| TCGA-B8-4622-01A | 1.43E-01 | 1.36E-01 | 2.02E-01 | 1.50E-01 | 1.65E-01 | | 5.54E-01 |
| TCGA-B8-5158-01A | 8.78E-02 | 1.35E-01 | 1.82E-01 | 2.26E-01 | 1.04E-01 | | 6.24E-01 |
| TCGA-B8-5163-01A | 2.18E-01 | 2.28E-01 | 4.36E-01 | 2.84E-01 | 2.66E-01 | | 1.01E+00 |
| TCGA-B8-5164-01A | 2.60E-01 | 1.87E-01 | 6.85E-01 | 3.50E-01 | 1.98E-01 | | 1.20E+00 |
| TCGA-B8-5550-01A | 1.35E-01 | 1.18E-01 | 1.28E-01 | 1.06E-01 | 1.42E-01 | | 5.74E-01 |
| TCGA-B8-A54D-01A | 1.62E-01 | 1.01E-01 | 4.50E-01 | 2.13E-01 | 1.46E-01 | | 5.67E-01 |
| TCGA-B9-5155-01A | 3.13E-02 | 8.63E-02 | 1.40E-01 | 8.94E-02 | 8.42E-02 | | 3.85E-01 |
| TCGA-B9-A44B-01A | 6.88E-02 | 7.14E-02 | 1.50E-01 | 7.98E-02 | 0.00E+00 | | 2.94E-01 |
| TCGA-B9-A69E-01A | 1.78E-01 | 1.61E-01 | 2.42E-01 | 1.40E-01 | 1.52E-01 | | 5.61E-01 |
| TCGA-BP-4160-01A | 1.87E-01 | 0.00E+00 | 6.56E-01 | 2.45E-01 | 8.90E-02 | | 8.34E-01 |
| TCGA-BP-4163-01A | 1.48E-01 | 1.32E-01 | 1.03E-01 | 1.70E-01 | 1.21E-01 | | 4.90E-01 |
| TCGA-BP-4166-01A | 4.94E-02 | 7.04E-02 | 2.11E-01 | 7.79E-02 | 4.58E-02 | | 4.08E-01 |
| TCGA-BP-4167-01A | 5.39E-02 | 1.53E-01 | 3.81E-01 | 1.44E-01 | 1.14E-01 | | 6.53E-01 |
| TCGA-BP-4329-01A | 9.02E-02 | 8.16E-02 | 1.69E-01 | 8.59E-02 | 1.73E-02 | | 4.65E-01 |
| TCGA-BP-4330-01A | 2.10E-01 | 2.90E-01 | 4.18E-01 | 2.04E-01 | 8.93E-02 | | 7.91E-01 |
| TCGA-BP-4332-01A | 5.77E-02 | 1.37E-01 | 1.43E-01 | 1.01E-01 | 7.64E-02 | | 3.70E-01 |
| TCGA-BP-4334-01A | 1.41E-04 | 3.98E-02 | 0.00E+00 | 0.00E+00 | 0.00E+00 | | 4.73E-03 |
| TCGA-BP-4335-01A | 6.02E-02 | 1.05E-01 | 2.02E-01 | 1.11E-01 | 0.00E+00 | | 3.53E-01 |
| TCGA-BP-4343-01A | 1.53E-01 | 2.06E-02 | 4.28E-01 | 1.88E-01 | 3.39E-01 | | 6.93E-01 |
| TCGA-BP-4345-01A | 1.13E-01 | 1.89E-01 | 2.33E-01 | 1.41E-01 | 1.16E-01 | | 7.14E-01 |
| TCGA-BP-4347-01A | 8.58E-02 | 1.65E-01 | 2.18E-01 | 9.85E-02 | 0.00E+00 | | 4.81E-01 |
| TCGA-BP-4351-01A | 2.54E-02 | 1.37E-01 | 1.08E-01 | 6.06E-02 | 1.45E-02 | | 3.13E-01 |
| TCGA-BP-4352-01A | 0.00E+00 | 1.42E-01 | 3.94E-02 | 0.00E+00 | 0.00E+00 | | 3.11E-01 |
| TCGA-BP-4354-01A | 0.00E+00 | 1.15E-01 | 2.94E-01 | 8.44E-01 | 8.76E-01 | | 9.41E-01 |
| TCGA-BP-4761-01A | 1.27E-01 | 7.52E-02 | 5.20E-02 | 2.75E-02 | 0.00E+00 | | 2.45E-01 |
| TCGA-BP-4770-01A | 0.00E+00 | 3.62E-02 | 2.93E-01 | 1.40E-01 | 2.74E-01 | | 6.03E-01 |
| TCGA-BP-4771-01A | 1.80E-01 | 2.98E-01 | 9.29E-01 | 3.31E-01 | 0.00E+00 | | 9.93E-01 |
| TCGA-BP-4787-01A | 6.41E-02 | 1.55E-01 | 4.10E-02 | 1.67E-01 | 2.85E-01 | | 4.63E-01 |
| TCGA-BP-4797-01A | 2.04E-01 | 1.23E-01 | 1.87E-01 | 1.34E-01 | 1.23E-01 | | 6.10E-01 |
| TCGA-BP-4799-01A | 1.66E-02 | 0.00E+00 | 2.44E-01 | 5.21E-02 | 0.00E+00 | | 4.25E-01 |
| TCGA-BP-4803-01A | 7.06E-02 | 5.70E-02 | 1.60E-01 | 8.76E-02 | 5.25E-02 | | 3.77E-01 |
| TCGA-BP-4967-01A | 1.09E-01 | 6.51E-02 | 5.71E-02 | 5.16E-02 | 4.47E-02 | | 3.31E-01 |
| TCGA-BP-4970-01A | 1.48E-01 | 2.11E-01 | 3.00E-01 | 2.48E-01 | 2.13E-01 | | 8.06E-01 |
| TCGA-BP-4971-01A | 7.74E-02 | 3.31E-01 | 0.00E+00 | 1.31E-01 | 0.00E+00 | | 5.52E-01 |
| TCGA-BP-4972-01A | 1.17E-01 | 1.05E-01 | 1.08E-01 | 1.82E-01 | 3.34E-01 | | 6.59E-01 |
| TCGA-BP-4973-01A | 1.01E-01 | 1.63E-01 | 1.18E-01 | 1.30E-01 | 3.41E-02 | | 4.20E-01 |
| TCGA-BP-4974-01A | 5.10E-02 | 2.81E-01 | 1.52E-01 | 1.52E-01 | 1.42E-02 | | 4.18E-01 |
| TCGA-BP-4983-01A | 1.22E-01 | 1.84E-01 | 6.22E-01 | 3.51E-01 | 1.57E-02 | | 1.13E+00 |
| TCGA-BP-4985-01A | 6.51E-02 | 8.62E-02 | 1.82E-01 | 1.23E-01 | 0.00E+00 | | 6.15E-01 |
| TCGA-BP-4989-01A | 1.58E-01 | 8.21E-02 | 6.80E-01 | 2.25E-01 | 0.00E+00 | | 7.96E-01 |
| TCGA-BP-5010-01A | 0.00E+00 | 1.80E-01 | 1.05E-02 | 3.59E-02 | 0.00E+00 | | 2.54E-01 |
| TCGA-BP-5178-01A | 1.15E-01 | 8.30E-02 | 8.31E-02 | 7.26E-02 | 6.54E-02 | | 3.66E-01 |
| TCGA-BP-5183-01A | 1.35E-01 | 9.80E-02 | 2.55E-01 | 1.26E-01 | 0.00E+00 | | 5.30E-01 |
| TCGA-BP-5191-01A | 6.61E-02 | 1.52E-01 | 1.94E-01 | 5.62E-02 | 0.00E+00 | | 3.40E-01 |
| TCGA-BP-5198-01A | 8.70E-02 | 1.10E-01 | 1.86E-01 | 1.79E-01 | 1.43E-01 | | 5.38E-01 |
| TCGA-BP-5201-01A | 8.65E-02 | 7.06E-02 | 4.48E-01 | 1.32E-01 | 0.00E+00 | | 4.64E-01 |
| TCGA-BP-5202-01A | 5.17E-02 | 1.13E-01 | 1.61E-01 | 1.14E-01 | 2.62E-02 | | 4.03E-01 |
| TCGA-BQ-5875-01A | 4.59E-02 | 9.09E-02 | 1.66E-01 | 1.09E-01 | 4.83E-04 | | 3.94E-01 |
| TCGA-BQ-5877-01A | 3.48E-01 | 1.41E-01 | 2.26E-01 | 1.52E-01 | 1.79E-01 | | 7.53E-01 |
| TCGA-BQ-5878-01A | 8.41E-02 | 1.11E-01 | 1.49E-01 | 1.05E-01 | 1.87E-02 | | 4.15E-01 |
| TCGA-BQ-5879-01A | 1.58E-01 | 1.05E-01 | 2.31E-01 | 1.61E-01 | 7.62E-02 | | 5.09E-01 |
| TCGA-BQ-5880-01A | 9.64E-02 | 1.22E-01 | 1.87E-01 | 9.11E-02 | 1.36E-02 | | 4.11E-01 |
| TCGA-BQ-5882-01A | 1.27E-01 | 1.98E-01 | 3.50E-01 | 1.03E-01 | 1.44E-01 | | 4.71E-01 |
| TCGA-BQ-5885-01A | 9.28E-02 | 1.27E-01 | 2.03E-01 | 9.97E-02 | 2.57E-02 | | 4.06E-01 |
| TCGA-BQ-5886-01A | 3.30E-02 | 6.23E-02 | 1.57E-01 | 7.74E-02 | 0.00E+00 | | 2.88E-01 |
| TCGA-BQ-5887-01A | 9.99E-02 | 1.54E-01 | 1.89E-01 | 1.19E-01 | 1.52E-01 | | 5.15E-01 |
| TCGA-BQ-5889-01A | 3.80E-01 | 4.30E-01 | 2.29E-01 | 1.90E-01 | 1.31E-01 | | 7.61E-01 |
| TCGA-BQ-5890-01A | 9.75E-02 | 9.09E-02 | 1.83E-01 | 9.92E-02 | 0.00E+00 | | 3.84E-01 |
| TCGA-BQ-5891-01A | 8.99E-02 | 7.56E-02 | 1.51E-01 | 8.21E-02 | 1.51E-02 | | 3.92E-01 |
| TCGA-BQ-5893-01A | 7.22E-02 | 1.14E-01 | 2.64E-01 | 8.54E-02 | 0.00E+00 | | 4.57E-01 |
| TCGA-BQ-5894-01A | 2.43E-01 | 1.99E-01 | 3.20E-01 | 1.21E-01 | 6.93E-02 | | 6.89E-01 |
| TCGA-BQ-7044-01A | 6.45E-02 | 8.03E-02 | 1.55E-01 | 8.47E-02 | 0.00E+00 | | 3.34E-01 |
| TCGA-BQ-7048-01A | 4.52E-02 | 1.80E-01 | 2.82E-01 | 6.82E-02 | 5.81E-02 | | 5.34E-01 |
| TCGA-BQ-7053-01A | 1.18E-01 | 1.28E-01 | 1.66E-01 | 1.05E-01 | 3.13E-03 | | 4.57E-01 |
| TCGA-BQ-7056-01A | 6.91E-02 | 8.10E-02 | 1.30E-01 | 8.64E-02 | 0.00E+00 | | 3.78E-01 |
| TCGA-BQ-7058-01A | 3.59E-02 | 5.54E-02 | 1.60E-01 | 7.12E-02 | 0.00E+00 | | 2.51E-01 |
| TCGA-CJ-4636-01A | 1.01E-01 | 1.21E-01 | 2.79E-01 | 1.71E-01 | 2.41E-01 | | 5.67E-01 |
| TCGA-CJ-4637-01A | 2.28E-01 | 2.67E-01 | 9.72E-01 | 4.03E-01 | 5.77E-02 | | 1.23E+00 |
| TCGA-CJ-4638-01A | 4.74E-02 | 5.44E-02 | 9.77E-03 | 0.00E+00 | 0.00E+00 | | 9.95E-02 |
| TCGA-CJ-4640-01A | 2.31E-01 | 8.10E-02 | 3.96E-01 | 1.90E-01 | 1.28E-01 | | 7.79E-01 |
| TCGA-CJ-4641-01A | 2.56E-01 | 3.18E-01 | 4.52E-01 | 2.53E-01 | 8.88E-02 | | 9.57E-01 |
| TCGA-CJ-4644-01A | 1.16E-01 | 5.16E-02 | 3.82E-01 | 1.54E-01 | 8.51E-02 | | 6.03E-01 |
| TCGA-CJ-4868-01A | 7.86E-02 | 1.62E-01 | 3.72E-01 | 2.13E-01 | 2.11E-01 | | 7.87E-01 |
| TCGA-CJ-4869-01A | 2.70E-01 | 1.32E-01 | 7.01E-01 | 1.86E-01 | 0.00E+00 | | 8.21E-01 |
| TCGA-CJ-4870-01A | 1.45E-02 | 1.04E-01 | 7.25E-02 | 4.54E-02 | 0.00E+00 | | 2.71E-01 |
| TCGA-CJ-4871-01A | 1.00E-01 | 9.83E-02 | 1.25E-01 | 8.88E-02 | 0.00E+00 | | 4.18E-01 |
| TCGA-CJ-4873-01A | 8.45E-02 | 1.79E-01 | 1.20E-01 | 8.98E-02 | 4.51E-02 | | 6.21E-01 |
| TCGA-CJ-4875-01A | 2.46E-02 | 3.90E-02 | 3.17E-01 | 5.83E-02 | 0.00E+00 | | 2.88E-01 |
| TCGA-CJ-4878-01A | 7.06E-02 | 1.49E-01 | 5.44E-02 | 9.05E-02 | 0.00E+00 | | 3.40E-01 |
| TCGA-CJ-4881-01A | 1.07E-01 | 1.75E-01 | 1.29E-01 | 1.67E-01 | 1.03E-01 | | 5.22E-01 |
| TCGA-CJ-4882-01A | 1.80E-01 | 4.25E-01 | 1.29E-01 | 1.60E-01 | 0.00E+00 | | 5.94E-01 |
| TCGA-CJ-4884-01A | 1.62E-01 | 1.50E-01 | 2.03E-01 | 1.28E-01 | 5.44E-02 | | 5.68E-01 |
| TCGA-CJ-4885-01A | 8.23E-02 | 3.55E-02 | 1.05E-01 | 5.94E-02 | 0.00E+00 | | 3.56E-01 |
| TCGA-CJ-4887-01A | 1.69E-01 | 9.04E-02 | 3.87E-01 | 1.49E-01 | 6.21E-02 | | 6.64E-01 |
| TCGA-CJ-4888-01A | 1.93E-01 | 5.89E-02 | 6.67E-01 | 3.97E-01 | 1.42E-01 | | 9.14E-01 |
| TCGA-CJ-4890-01A | 6.47E-02 | 2.75E-01 | 5.31E-01 | 3.59E-01 | 5.14E-01 | | 8.61E-01 |
| TCGA-CJ-4894-01A | 1.86E-01 | 1.40E-01 | 4.76E-01 | 2.06E-01 | 2.73E-02 | | 8.20E-01 |
| TCGA-CJ-4895-01A | 5.10E-02 | 1.59E-01 | 1.91E-01 | 2.08E-01 | 2.07E-01 | | 5.64E-01 |
| TCGA-CJ-4897-01A | 1.31E-01 | 7.72E-02 | 1.29E-01 | 5.34E-02 | 0.00E+00 | | 4.05E-01 |
| TCGA-CJ-4901-01A | 1.43E-01 | 2.24E-01 | 5.20E-01 | 2.52E-01 | 5.87E-03 | | 7.94E-01 |
| TCGA-CJ-4902-01A | 9.57E-02 | 1.71E-01 | 2.73E-01 | 1.40E-01 | 1.49E-01 | | 5.62E-01 |
| TCGA-CJ-4904-01A | 5.51E-02 | 1.71E-01 | 2.86E-01 | 1.75E-01 | 7.86E-02 | | 5.34E-01 |
| TCGA-CJ-4907-01A | 1.26E-01 | 1.11E-01 | 1.75E-01 | 1.01E-01 | 8.77E-02 | | 5.14E-01 |
| TCGA-CJ-4916-01A | 7.16E-02 | 9.30E-02 | 7.72E-01 | 2.30E-01 | 0.00E+00 | | 7.16E-01 |
| TCGA-CJ-4918-01A | 6.76E-02 | 1.84E-01 | 2.08E-01 | 1.56E-01 | 1.49E-01 | | 5.52E-01 |
| TCGA-CJ-5676-01A | 5.12E-02 | 1.47E-01 | 1.91E-01 | 1.14E-01 | 6.69E-02 | | 4.13E-01 |
| TCGA-CJ-5677-01A | 1.08E-01 | 8.28E-02 | 5.37E-02 | 1.13E-01 | 1.02E-01 | | 5.53E-01 |
| TCGA-CJ-5678-01A | 2.74E-01 | 7.67E-03 | 3.96E-01 | 1.20E-01 | 0.00E+00 | | 7.31E-01 |
| TCGA-CJ-5679-01A | 0.00E+00 | 3.48E-02 | 3.38E-02 | 4.32E-03 | 0.00E+00 | | 2.94E-01 |
| TCGA-CJ-5680-01A | 1.30E-01 | 1.01E-01 | 4.01E-02 | 7.81E-02 | 0.00E+00 | | 4.80E-01 |
| TCGA-CJ-5681-01A | 1.76E-01 | 1.71E-01 | 0.00E+00 | 1.62E-02 | 7.04E-02 | | 5.97E-01 |
| TCGA-CJ-5682-01A | 7.70E-02 | 6.06E-02 | 2.38E-01 | 9.56E-02 | 0.00E+00 | | 4.49E-01 |
| TCGA-CJ-5684-01A | 1.80E-01 | 4.36E-02 | 4.01E-01 | 7.87E-02 | 0.00E+00 | | 5.64E-01 |
| TCGA-CJ-6028-01A | 2.10E-01 | 1.79E-01 | 5.51E-01 | 2.97E-01 | 1.53E-01 | | 8.35E-01 |
| TCGA-CJ-6033-01A | 1.13E-01 | 1.27E-01 | 1.27E-01 | 7.21E-02 | 4.42E-02 | | 4.96E-01 |
| TCGA-CW-5584-01A | 1.45E-01 | 1.79E-01 | 5.04E-02 | 1.54E-01 | 9.42E-02 | | 5.31E-01 |
| TCGA-CW-5585-01A | 1.01E-01 | 9.97E-02 | 8.13E-02 | 4.13E-02 | 0.00E+00 | | 3.73E-01 |
| TCGA-CW-5587-01A | 8.76E-02 | 1.69E-01 | 5.70E-01 | 3.21E-01 | 1.36E-01 | | 8.24E-01 |
| TCGA-CW-5590-01A | 7.22E-02 | 1.16E-01 | 2.31E-01 | 1.17E-01 | 0.00E+00 | | 4.62E-01 |
| TCGA-CW-5591-01A | 1.31E-01 | 1.71E-01 | 2.38E-02 | 8.06E-02 | 1.11E-01 | | 4.32E-01 |
| TCGA-CW-6097-01A | 2.05E-01 | 4.76E-01 | 2.01E-01 | 2.75E-01 | 2.46E-01 | | 8.38E-01 |
| TCGA-CZ-4857-01A | 6.62E-02 | 1.63E-01 | 6.60E-02 | 1.31E-01 | 1.59E-01 | | 5.10E-01 |
| TCGA-CZ-4863-01A | 2.16E-01 | 1.67E-01 | 5.68E-01 | 2.49E-01 | 0.00E+00 | | 8.70E-01 |
| TCGA-CZ-5454-01A | 8.22E-02 | 0.00E+00 | 1.90E-01 | 1.60E-01 | 2.74E-01 | | 6.82E-01 |
| TCGA-CZ-5457-01A | 1.52E-01 | 3.71E-02 | 2.09E-01 | 7.94E-02 | 5.24E-02 | | 4.96E-01 |
| TCGA-CZ-5458-01A | 7.41E-02 | 1.71E-01 | 1.98E-01 | 1.38E-01 | 7.85E-02 | | 4.46E-01 |
| TCGA-CZ-5459-01A | 6.29E-02 | 1.05E-01 | 4.51E-02 | 6.70E-02 | 0.00E+00 | | 2.86E-01 |
| TCGA-CZ-5460-01A | 1.32E-01 | 1.34E-01 | 1.01E-01 | 1.06E-01 | 0.00E+00 | | 4.07E-01 |
| TCGA-CZ-5461-01A | 1.22E-01 | 1.35E-01 | 1.23E-01 | 1.61E-01 | 1.07E-01 | | 5.73E-01 |
| TCGA-CZ-5464-01A | 1.58E-01 | 1.60E-01 | 2.46E-01 | 1.63E-01 | 3.85E-02 | | 6.50E-01 |
| TCGA-CZ-5466-01A | 1.99E-01 | 1.78E-01 | 6.14E-02 | 9.18E-02 | 4.80E-02 | | 7.28E-01 |
| TCGA-CZ-5467-01A | 6.33E-02 | 1.18E-01 | 1.29E-01 | 8.66E-02 | 2.56E-02 | | 4.19E-01 |
| TCGA-CZ-5987-01A | 9.06E-02 | 1.15E-01 | 1.14E-01 | 1.25E-01 | 2.03E-03 | | 4.65E-01 |
| TCGA-EU-5907-01A | 1.58E-01 | 7.18E-02 | 0.00E+00 | 6.96E-02 | 0.00E+00 | | 3.38E-01 |
| TCGA-F9-A4JJ-01A | 1.29E-01 | 1.51E-01 | 3.46E-01 | 9.51E-02 | 0.00E+00 | | 5.40E-01 |
| TCGA-F9-A8NY-01A | 2.01E-01 | 1.02E-01 | 3.26E-01 | 4.98E-02 | 0.00E+00 | | 5.67E-01 |
| TCGA-F9-A97G-01A | 4.18E-02 | 1.47E-01 | 1.70E-01 | 6.96E-02 | 2.91E-01 | | 6.90E-01 |
| TCGA-G6-A8L6-01A | 4.54E-02 | 1.38E-01 | 8.76E-02 | 9.14E-02 | 0.00E+00 | | 3.68E-01 |
| TCGA-G7-6797-01A | 3.87E-02 | 1.22E-01 | 1.23E-01 | 9.31E-02 | 5.15E-03 | | 4.59E-01 |
| TCGA-G7-7501-01A | 7.26E-02 | 9.24E-02 | 1.69E-01 | 9.63E-02 | 8.13E-02 | | 4.30E-01 |
| TCGA-G7-A8LB-01A | 2.86E-02 | 1.12E-01 | 1.08E-01 | 7.30E-02 | 0.00E+00 | | 2.59E-01 |
| TCGA-G7-A8LD-01A | 1.29E-01 | 1.17E-01 | 2.56E-01 | 1.05E-01 | 2.63E-02 | | 4.61E-01 |
| TCGA-GL-6846-01A | 4.96E-02 | 1.17E-01 | 1.32E-01 | 8.65E-02 | 0.00E+00 | | 4.08E-01 |
| TCGA-GL-7966-01A | 5.72E-02 | 1.27E-01 | 2.50E-01 | 1.04E-01 | 0.00E+00 | | 5.14E-01 |
| TCGA-GL-A59R-01A | 5.55E-02 | 1.40E-01 | 1.26E-01 | 1.07E-01 | 7.22E-02 | | 4.30E-01 |
| TCGA-HE-7130-01A | 5.53E-02 | 1.68E-01 | 2.24E-01 | 1.80E-01 | 0.00E+00 | | 4.55E-01 |
| TCGA-IA-A40U-01A | 9.65E-02 | 1.30E-01 | 2.14E-01 | 1.38E-01 | 6.37E-02 | | 4.38E-01 |
| TCGA-IA-A40Y-01A | 8.47E-02 | 1.00E-01 | 1.89E-01 | 1.15E-01 | 3.75E-02 | | 4.62E-01 |
| TCGA-J7-8537-01A | 2.08E-01 | 2.34E-01 | 3.12E-01 | 1.32E-01 | 1.65E-01 | | 6.96E-01 |
| TCGA-KL-8323-01A | 8.71E-02 | 1.01E-01 | 2.11E-01 | 1.16E-01 | 3.76E-02 | | 4.51E-01 |
| TCGA-KL-8326-01A | 7.79E-02 | 1.01E-01 | 2.08E-01 | 1.10E-01 | 4.21E-02 | | 4.29E-01 |
| TCGA-KL-8334-01A | 8.97E-02 | 1.08E-01 | 2.09E-01 | 1.17E-01 | 5.53E-02 | | 4.72E-01 |
| TCGA-KL-8335-01A | 8.64E-02 | 1.03E-01 | 2.00E-01 | 1.15E-01 | 1.45E-02 | | 4.49E-01 |
| TCGA-KL-8336-01A | 9.45E-02 | 1.09E-01 | 2.06E-01 | 1.19E-01 | 3.83E-02 | | 4.83E-01 |
| TCGA-KL-8338-01A | 9.02E-02 | 1.04E-01 | 2.04E-01 | 1.09E-01 | 2.86E-02 | | 4.69E-01 |
| TCGA-KL-8339-01A | 1.02E-01 | 1.01E-01 | 2.05E-01 | 1.22E-01 | 1.66E-03 | | 4.76E-01 |
| TCGA-KL-8341-01A | 9.81E-02 | 9.04E-02 | 2.13E-01 | 1.19E-01 | 1.94E-02 | | 4.57E-01 |
| TCGA-KL-8344-01A | 1.39E-01 | 1.14E-01 | 2.36E-01 | 1.20E-01 | 7.33E-02 | | 5.14E-01 |
| TCGA-KL-8345-01A | 8.27E-02 | 1.02E-01 | 2.06E-01 | 1.13E-01 | 5.09E-02 | | 4.50E-01 |
| TCGA-KM-8440-01A | 8.66E-02 | 1.04E-01 | 2.04E-01 | 1.14E-01 | 8.20E-03 | | 4.53E-01 |
| TCGA-KN-8426-01A | 1.03E-01 | 1.18E-01 | 1.98E-01 | 1.20E-01 | 2.13E-02 | | 4.84E-01 |
| TCGA-KN-8427-01A | 8.72E-02 | 1.53E-01 | 2.58E-01 | 1.11E-01 | 1.71E-01 | | 6.17E-01 |
| TCGA-KN-8429-01A | 1.30E-01 | 8.85E-02 | 2.21E-01 | 1.11E-01 | 1.38E-02 | | 4.99E-01 |
| TCGA-KN-8433-01A | 8.75E-02 | 1.26E-01 | 1.83E-01 | 1.16E-01 | 3.50E-02 | | 4.59E-01 |
| TCGA-KO-8404-01A | 7.99E-02 | 2.21E-01 | 1.73E-01 | 1.57E-01 | 2.52E-01 | | 5.98E-01 |
| TCGA-KO-8405-01A | 1.45E-01 | 1.12E-01 | 1.94E-01 | 1.09E-01 | 1.03E-01 | | 6.32E-01 |
| TCGA-KO-8408-01A | 8.41E-02 | 1.12E-01 | 1.91E-01 | 1.16E-01 | 1.17E-02 | | 4.56E-01 |
| TCGA-KO-8416-01A | 8.22E-02 | 1.09E-01 | 1.99E-01 | 1.12E-01 | 3.82E-02 | | 4.55E-01 |
| TCGA-P4-A5E6-01A | 6.36E-02 | 1.34E-01 | 1.54E-01 | 9.48E-02 | 9.01E-03 | | 3.69E-01 |
| TCGA-P4-A5E8-01A | 7.21E-02 | 1.06E-01 | 3.18E-01 | 9.12E-02 | 0.00E+00 | | 5.33E-01 |
| TCGA-P4-A5EA-01A | 4.77E-01 | 4.38E-01 | 4.41E-01 | 1.71E-01 | 0.00E+00 | | 6.99E-01 |
| TCGA-P4-AAVK-01A | 8.93E-02 | 6.93E-02 | 1.69E-01 | 8.48E-02 | 4.40E-02 | | 4.14E-01 |
| TCGA-P4-AAVL-01A | 6.31E-02 | 1.64E-01 | 1.41E-01 | 1.21E-01 | 2.20E-02 | | 4.61E-01 |
| TCGA-Q2-A5QZ-01A | 9.47E-02 | 1.29E-01 | 1.39E-01 | 8.97E-02 | 3.58E-02 | | 4.44E-01 |
| TCGA-UZ-A9PN-01A | 1.55E-01 | 2.48E-01 | 1.49E-01 | 1.15E-01 | 1.65E-02 | | 5.76E-01 |
| TCGA-UZ-A9PQ-01A | 5.08E-02 | 6.59E-02 | 1.63E-01 | 8.54E-02 | 0.00E+00 | | 3.13E-01 |
| TCGA-UZ-A9PZ-01A | 6.16E-02 | 1.37E-01 | 1.25E-01 | 1.04E-01 | 0.00E+00 | | 3.75E-01 |
| TCGA-Y8-A896-01A | 4.75E-01 | 2.92E-01 | 1.94E-01 | 1.59E-01 | 1.36E-01 | | 9.84E-01 |
